# Proteomic analysis identifies subgroups of patients with active systemic lupus erythematosus

**DOI:** 10.1186/s12014-023-09420-1

**Published:** 2023-07-29

**Authors:** Kevin Y. C. Su, John A. Reynolds, Rachel Reed, Rachael Da Silva, Janet Kelsall, Ivona Baricevic-Jones, David Lee, Anthony D. Whetton, Nophar Geifman, Neil McHugh, Ian N. Bruce, the MASTERPLANS and BILAG-BR consortia

**Affiliations:** 1 Rheumatology Research Group, Institute of Inflammation and Ageing, University of Birmingham, Birmingham, UK; 2 Rheumatology Department, Sandwell and West Birmingham NHS Trust, Birmingham, UK; 3 Stoller Biomarker Discovery Centre, Division of Cancer Sciences, Faculty of Biology, Medicine and Health, University of Manchester, Manchester, UK; 4 Faculty of Health and Medical Sciences, University of Surrey, Guildford, UK; 5 Department of Pharmacy and Pharmacology, University of Bath, Bath, UK; 6 Centre for Epidemiology Versus Arthritis, Division of Musculoskeletal and Dermatological Sciences, The University of Manchester, Manchester, UK; 7 NIHR Manchester Biomedical Research Centre, Manchester University Hospitals NHS Foundation Trust, Manchester Academic Health Science Centre, Manchester, UK

**Keywords:** SLE (systemic lupus erythematosus), SWATH, Proteomics, Cluster, Arthritis, Nephritis

## Abstract

**Objective:**

Systemic lupus erythematosus (SLE) is a clinically and biologically heterogenous autoimmune disease. We aimed to investigate the plasma proteome of patients with active SLE to identify novel subgroups, or endotypes, of patients.

**Method:**

Plasma was collected from patients with active SLE who were enrolled in the British Isles Lupus Assessment Group Biologics Registry (BILAG-BR). The plasma proteome was analysed using a data-independent acquisition method, Sequential Window Acquisition of All theoretical mass spectra mass spectrometry (SWATH-MS). Unsupervised, data-driven clustering algorithms were used to delineate groups of patients with a shared proteomic profile.

**Results:**

In 223 patients, six clusters were identified based on quantification of 581 proteins. Between the clusters, there were significant differences in age (p = 0.012) and ethnicity (p = 0.003). There was increased musculoskeletal disease activity in cluster 1 (C1), 19/27 (70.4%) (p = 0.002) and renal activity in cluster 6 (C6) 15/24 (62.5%) (p = 0.051). Anti-SSa/Ro was the only autoantibody that significantly differed between clusters (p = 0.017). C1 was associated with p21-activated kinases (PAK) and Phospholipase C (PLC) signalling. Within C1 there were two sub-clusters (C1A and C1B) defined by 49 proteins related to cytoskeletal protein binding. C2 and C6 demonstrated opposite Rho family GTPase and Rho GDI signalling. Three proteins (MZB1, SND1 and AGL) identified in C6 increased the classification of active renal disease although this did not reach statistical significance (p = 0.0617).

**Conclusions:**

Unsupervised proteomic analysis identifies clusters of patients with active SLE, that are associated with clinical and serological features, which may facilitate biomarker discovery. The observed proteomic heterogeneity further supports the need for a personalised approach to treatment in SLE.

**Supplementary Information:**

The online version contains supplementary material available at 10.1186/s12014-023-09420-1.

## Introduction

Systemic lupus erythematosus (SLE) is an inflammatory autoimmune rheumatic disease with significant morbidity and mortality [1]; it is heterogeneous in both clinical features and treatment response. Although several new treatments for SLE have been developed, there is an important unmet need for disease biomarkers to measure active disease and predict treatment response.

The clinical heterogeneity of SLE may reflect different underlying cellular and molecular processes. Despite a better understanding of mechanisms that contribute to disease such as increased B cell activity and type 1 interferon production, which have led to more targeted novel therapies, the overall response rates to these therapies in clinical trials are typically around 40–60% [2, 3]. A more personalised approach to therapy may increase response rates. For example, a post hoc analysis of randomised controlled trials using belimumab (BEL), an anti-BAFF monoclonal antibody, identified increased efficacy in patients with elevated anti-dsDNA antibody or low C3/4 complement, suggesting different patient subgroups may have different treatment response [4]. Common biomarkers have variable sensitivity and specificity for active disease (approximately 62% and 93% for anti-dsDNA antibodies, 75% and 71% for C3 complements, and 48% and 71% for C4 complements) [5]. The UK Medical Research Council (MRC) Precision Medicine Consortium ‘Maximising SLE Therapeutic Potential by the Application of Novel and Stratified Approaches’ (MASTERPLANS) aimed to identify novel markers to predict treatment response. An important first step was to identify biomarkers which may associate with specific clinical features of SLE.

Proteomic analysis using mass-spectrometry (MS) has been applied in numerous immune-mediated inflammatory diseases including Rheumatoid arthritis (RA) [6], Sjogren’s syndrome [7] and Systemic sclerosis [8]. Although proteomic MS studies in SLE to date have been modest in size, a systematic review by Nicolaou et al. identified 241 potential biomarkers from 25 studies using gel electrophoresis or liquid chromatography tandem MS across numerous sample types [9, 10] within different manifestations of SLE such as nephritis, neuropsychiatric and cutaneous disease. Of note, Annexin A2 which was observed in three studies included in the review, was not detected at a high level in our study and thus excluded from analysis.

Sequential window acquisition of all theoretical mass spectra (SWATH-MS) is a data independent acquisition (DIA) MS technique. It has advantages over data-dependant acquisition (DDA) and targeted acquisition (TA) methods as it employs non-selective analysis of peptides and their fragments. These data are then compared to a spectral library of protein fragments to deconvolve the complex signal into relative quantities of peptides and proteins across hundreds of samples [11]. DIA has high reproducibility and can detect peptides in the order of tens of thousands compared to TA, where typically hundreds of peptides are measured in a directed non-discovery approach [12]. Due to its unbiased nature and ability to identify and quantify peptides at the proteome-scale; SWATH-MS is a powerful method for biomarker discovery.

The aim of the study was to use SWATH-MS to determine whether analysis of the plasma proteome can identify discrete subgroups in a large cohort of SLE patients with highly active disease.

## Methods

### Study population

We included patients with SLE fulfilling the 1997 Updated American College of Rheumatology (ACR) or the Systemic Lupus International Collaborating Clinics (SLICC) 2012 classification criteria registered with the BILAG-Biologics Registry (BILAG-BR) [13]. All had active disease and due to commence rituximab (RTX), belimumab (BEL) or mycophenolate mofetil (MMF). Patients commencing RTX or BEL needed to have sufficiently active disease to satisfy the NHS England 2013 Interim Clinical Commissioning Policy and National Institute for Health and Care Excellence (NICE) criteria respectively [14, 15].

Baseline plasma samples were obtained from study patients within a window of maximum 30 days prior to and a maximum of five days after, receiving escalating treatment with BEL, RTX or MMF.

Baseline disease activity was measured using BILAG-2004 index [16] and SLEDAI-2000 (SLEDAI-2 K) [17]. Active disease in each domain was defined as BILAG-2004 A or B score. Lupus-related damage was measured using the ACR/SLICC damage index (SDI) [18]. Measurement of C3 and C4 levels were conducted at recruiting sites with low C3/C4 status determined by local laboratory reference ranges. Proteinuria was measured at the local site by spot urine protein-creatinine ratio (uPCR) or albumin-creatinine ratio (uACR). Values for uPCR were converted to uACR using the method described by Sumida et al. [19].

### Antibody status

All sera were tested for autoantibodies at the same centre by immunoprecipitation of proteins from radiolabelled cell lines, followed by PAGE separation and identification of recognised autoantigens (e.g. Ro60 [SS-A], U1RNP/Sm, La [SS-B]) by autoradiography as described elsewhere [20]. Anti-Ro52 was measured using the ABNOVA SS-A 52 Ab ELISA Kit and anti-dsDNA using the Inova Diagnostics Quanta Lite® dsDNA SC ELISA kit.

### Sample preparation

Plasma analysis was performed using Sequential Window Acquisition of All Theoretical Mass Spectra (SWATH-MS). Briefly, 10uL of plasma was depleted of highly abundant proteins (albumin, IgG, transferrin, fibrinogen, IgA, α-2-macroglobulin, α-1-antitrypsin, IgM, haptoglobin, α1-acid glycoprotein, apolipoprotein A-I and apolipoprotein A-II), then concentrated through centrifugal filtration and added to digestion buffer of ammonium bicarbonate, yielding a final volume of 80–100 uL. Proteins were reduced and solubilised with dithiothreitol and sodium deoxycholate then alkylated with iodoacetamide and digested with trypsin. The sample was recovered through centrifugation and peptides were lyophilised from recovered supernatant by vacuum centrifugation (MiVac Quattro Concentrator, SP Scientific US) and stored at − 80 °C until use (details in Additional file 1). Samples were analysed by SWATH-MS with a micro-flow LC–MS system comprising an Eksigent nanoLC 400 autosampler and an Eksigent nanoLC 425 pump coupled to a Sciex 6600 Triple-TOF mass spectrometer with a DuoSpray Ion Source. The system was controlled by Analyst software v1.7.1 and eksigent control software v4.2. This spectral library was generated on the basis of a previously published plasma protein library [21] but updated to be compatible with a 100 variable window acquisition method employed in the Stoller Centre. Full MS methodology is included in the Additional file 1.

SWATH maps were aligned with TRIC (*msproteomicstools* version 0.4.3) feature alignment algorithm. The aligned openSWATH maps were processed with *MSstats* to infer protein-level quantification based on aligned transition-level quantitative information. With this technique, proteins in low quantities, below the threshold of detection within the plasma, were designated as absent. Full methodology for alignment is included in the Additional file 1.

### Data analysis

Hierarchical clustering was used to cluster patients based on their proteomic profile. The Average linkage method was applied. The optimal number of clusters was determined using the R package, *nbclust* [22]. The elbow method was used to visually confirm the number of clusters, and the final number of clusters resolved by majority rule of 30 different validation indices (see Additional file 1). Initial data visualisation was performed on untransformed data using t-distributed stochastic neighbour embedding (t-SNE) with a perplexity of 11 (approximately 5% of the study population) [23]. For heat map visualisation, protein expression levels were standardised to Z scores (where if expression level = x, Z score calculated as (x – mean)/standard deviation).

Differential protein levels between clusters were performed with the proteome of a single cluster compared to the remaining proteome as a “combined cluster”, i.e., Cluster 1 vs all remaining. The comparison was performed using a linear model with multiple t-tests (R package *limma*) and an adjusted-P < 0.05 (Benjamini-Hochberg) was considered statistically significant. Canonical pathways and functional protein association networks were visualised using String (v.11.0, URL: https://string-db.org/) [24] and Qiagen Ingenuity Pathway Analysis (IPA) [25]. Pathways with Z score of < − 1 or > 1 was considered significant.

Clinical variables across clusters were analysed using non-parametric tests; Chi square with Fisher’s exact test and Kruskal–Wallis as appropriate. Those features which are considered increased/or decreased between clusters are reported based on descriptive numerical differences. In the regression models, finite mixture models were used to allocate proteins with a clear bimodal distribution into undetectable/low/high groups. Other proteins were considered binary (undetectable/detectable). An adaptive lasso regression model (with sample split into training and validation datasets at a ratio of 3:1) was used to identify proteins with a non-zero coefficient with lambda selected via cross-validation. Analyses were performed using R v.4.0.3, STATA v16.0 SE and SPSS v.26.

## Results

### Baseline patient characteristics

Plasma samples were analysed from 223 patients, 198 (88.8%) were females with median (IQR) age and disease duration of 40 (30, 51) and 10 (6, 17) years respectively. At baseline, 204 (91.5%) had at least one BILAG A and/or two BILAG B scores. Of these, active disease (BILAG 2004 A or B score) was predominantly in mucocutaneous (107, 51.2%), musculoskeletal (95, 45.5%) and renal (79, 37.4%) domains. Almost 40% had elevated anti-dsDNA antibodies (87/223 [39%]) and/or low C3 or C4 complement (100/223 [44.8%]).

Regarding treatment, 136/223 (61%) were taking regular oral corticosteroids with a median (IQR) dose of 10 (5, 14) mg/day. Almost half were taking an anti-malarial (AM) at the time of plasma acquisition (110/223 [49.3%]) although 202/223 (90.6%) had ever taken an AM and 77 (34.5%) were taking mycophenolate mofetil. The cohort demographics are shown in Table 1.

**Table 1:** Patient characteristics

Total (n = 223)	No (%)/median (IQR)
Age, years (n = 197)	40 (30, 51)
Female	198 (88.8)
Caucasian (n = 222)	132 (59.2)
Current smoker	27 (12.1)
Disease duration, years (n = 218)	10 (6, 17)
SLICC damage index (n = 206)	0 (0,1)
1997 ACR criteria at baseline (n = 203)	
Number fulfilling ≥ 4 criteria	186 (83.4)
Malar rash	122 (54.7)
Photosensitivity	115 (51.6)
Discoid rash	41 (18.4)
Oral ulcers	136 (61)
Arthritis	192 (86.1)
Serositis	69 (30.9)
CNS	22 (9.9)
Renal disease	81 (36.3)
Haematologic disorder	115 (51.6)
Immunologic disorder	151 (67.7)
Positive ANA	185 (83)
Disease activity	
SLEDAI score (n = 212)	8 (4.5, 14)
SLICC damage index (n = 206)	0 (0,1)
BILAG-2004 score at baseline	
Constitutional	19 (9.3)
Mucocutaneous	107 (51.2)
Neuropsychiatric	23 (11.2)
Musculoskeletal	95 (45.5)
Cardiorespiratory	35 (17)
Gastrointestinal	11 (5.4)
Ophthalmic	13 (6.4)
Renal	79 (37.4)
Haematological	9 (4.4)
Baseline creatinine, umol/l (n = 162)	66 (57, 78)
BMI, kg/m^2^ (n = 171)	26.4 (22.9, 31.3)
Medications	
Current steroid use	136 (61)
Usual daily OCS dose (mg/day)	10 (5, 14)
Current anti-malarial use	110 (49.3)
Anti-malarial use ever	202 (90.6)
Methotrexate	9 (4)
Azathioprine	11 (4.9)
Mycophenolate mofetil	77 (34.5)
Calcineurin inhibitor	3(1.3)
Serology	
Ro (n = 215)	76 (35.3)
Ro52 (n = 215)	42 (18.8)
Ro60 (n = 192)	70 (31.4)
La (n = 195)	19 (8.5)
dsDNA (n = 215)	87 (39)
dsDNA titre, IU/l	38.5 (13.9, 246.1)
U1-RNP (n = 192)	56 (25.1)
Low C3/C4 (n = 212)	100 (44.8)

*IQR* interquartile range, *dsDNA* Double stranded DNA, *CNS* Central Nervous System, *ANA* Anti-nuclear antibody, *SLEDAI* Systemic Lupus Erythematosus Disease Activity Index, *SLICC* Systemic Lupus International Collaborating Clinics, *BILAG* British Isles Lupus Assessment Group, *BMI* Body Mass Index, *OCS* Oral corticosteroids, *ACR* American College of Rheumatology.*U1-RNP* U1-Ribonuclear Protein, *MTX* Methotrexate, *AZA* Azathioprine, *MMF* Mycophenolate Mofetil, *CyA* Cyclosporin A, *TAC* Tacrolimus

### Cluster analysis

A total of 894 proteins were quantified by SWATH-MS. After removing proteins which were only detectable in < 25% of all samples, 588 remained. Seven more were removed due to high relative levels (> 20) with low variance (< 1), resulting in 581 proteins for analysis.

**Fig. 1:**
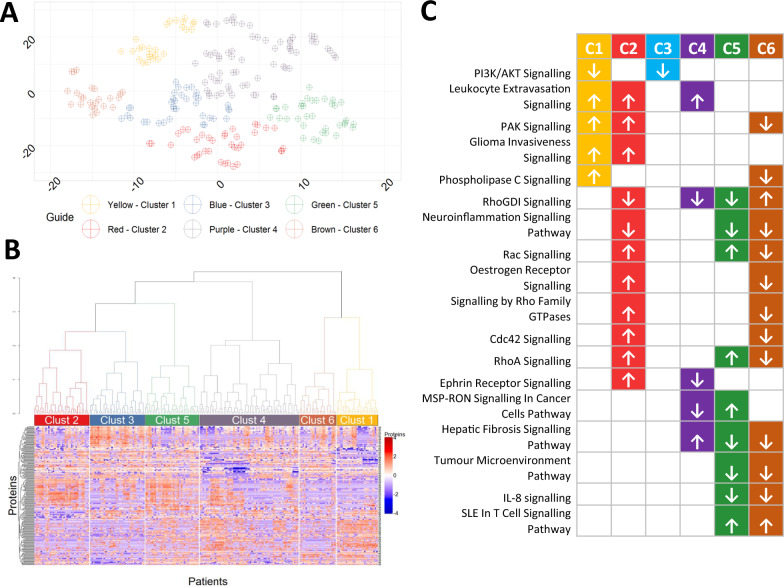
Cluster analysis of proteins in 223 patients with active SLE. **A** t-distributed stochastic neighbour embedding (t-SNE) plot of the 6 clusters following hierarchal clustering. Each point represents a single patient allocated to one of n = 6 clusters based on the plasma proteome alone. **B** Heatmap of the 6 clusters showing only those proteins which were significantly different in at least 1 cluster. Colour shows the Z-score with increased levels in red and decreased levels in blue. **C** Canonical pathways that are predicted to be activated or supressed in more than one cluster. The arrows show the direction (up = activation, down = suppression)

The plurality (11/30) of clustering algorithms suggested the data could be split into six clusters. Clusters were created using hierarchical clustering and visualised with t-SNE (Fig. 1). The largest cluster comprised 65 patients (29.1% of the patient cohort; cluster 4, C4) followed by 27 patients (12.1% of the cohort, cluster 1, C1), 27 patients (12.1% of the cohort, cluster 5, C5), 36 patients (16.1%, both cluster 2 and 3, C2 and C3) and the smallest comprised 24 patients (10.7% of the cohort; cluster 6, C6).

### Antibody status and age significantly differs between clusters

The youngest patients were in C6 (median age 30.5 [19, 40.25] years) and the oldest in C3 (45 [39.25, 50.5] years) (p = 0.012 between clusters). C6 had the least proportion of Caucasian patients (7/24 [29.2%]); for further description of ethnic groups see Additional file 1. Between clusters, there was no statistical difference in disease duration or SDI scores (Table 2).

**Table 2:**
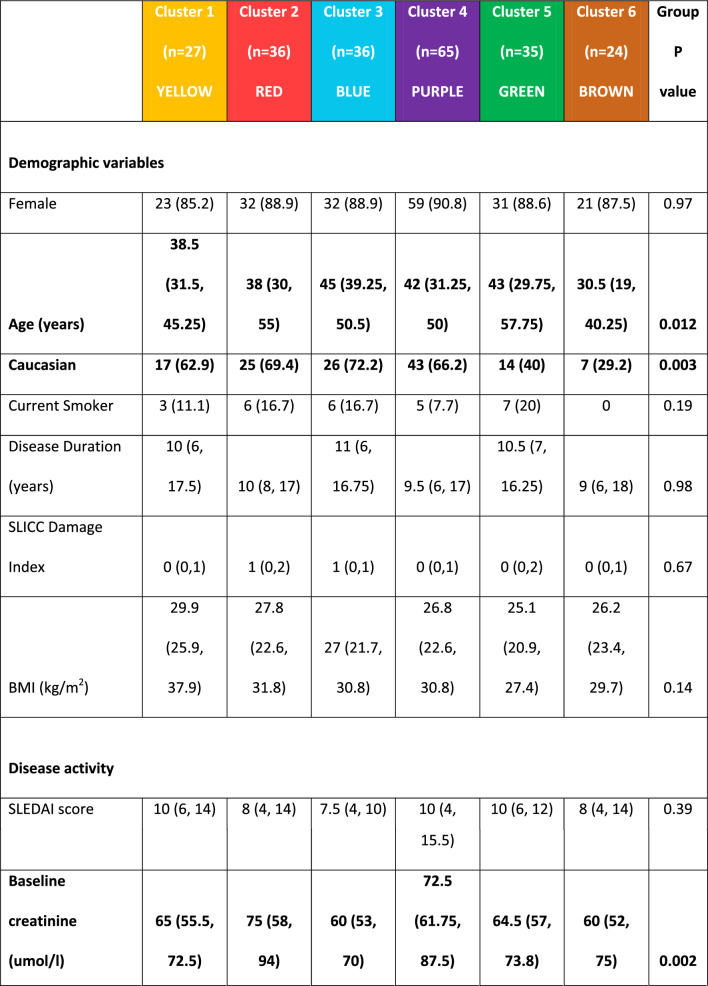

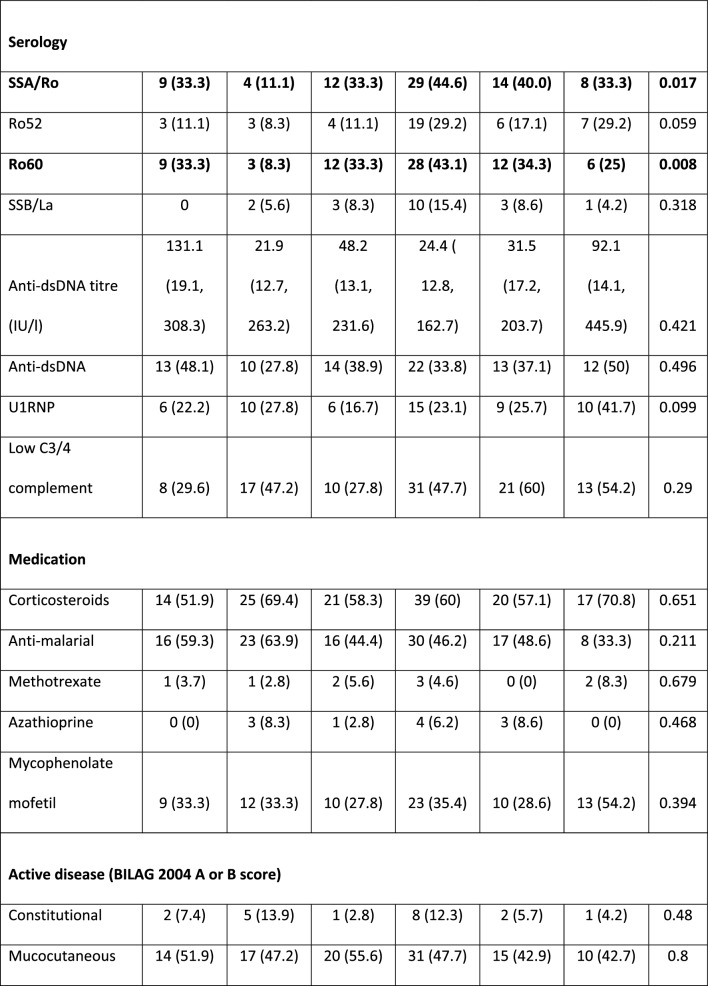

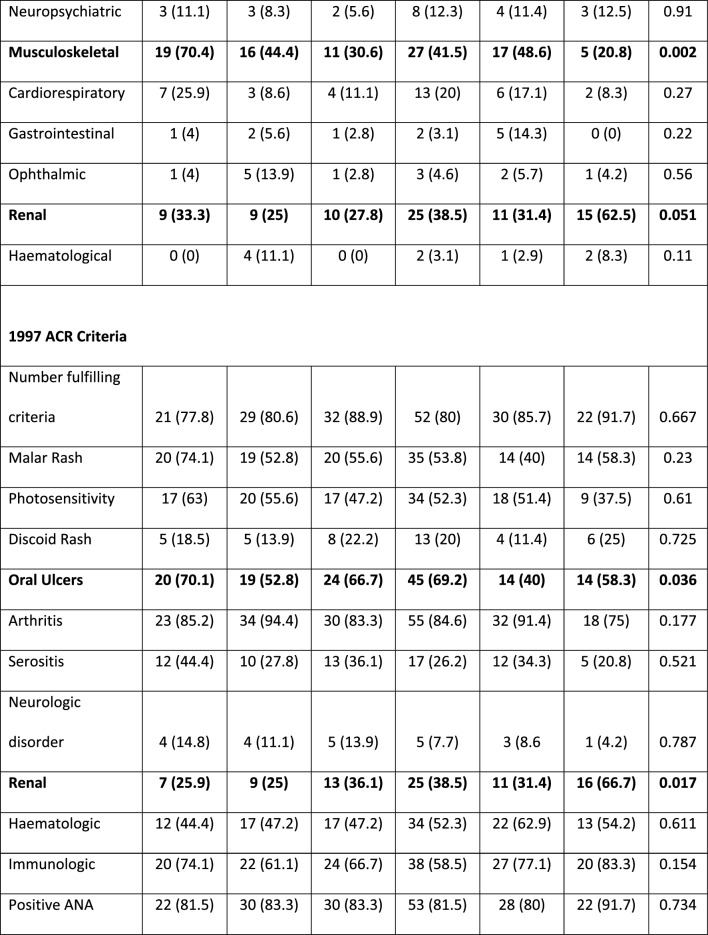
Comparison of patient characteristics between the 6 proteomic clusters

Values are n (%) or median (IQR) as appropriate. Comparisons between clusters were made using the Kruskal–Wallis test or Chi-2 test for continuous and categorical variables respectively

*BMI* Body Mass Index, *dsDNA* Double stranded DNA, *SLEDAI* Systemic Lupus Erythematosus Disease Activity Index, *SLICC* Systemic Lupus International Collaborating Clinics, *BILAG* British Isles Lupus Assessment Group, *ACR* American College of Rheumatology, *ANA* Anti-nuclear antibody, *U1-RNP* U1-Ribonuclear Protein

There was a significant difference in anti-SSA/Ro antibody positivity between clusters (p = 0.017), C4 had the highest frequency of anti-SSA/Ro (29/65 [44.6%]) and the lowest frequency was in C2 (4/36 [11.1%]). Similarly, anti-Ro60 was highest in C4, (28/65 [43.1%]) and lowest in C2 (3/36 [8.3%]) (p = 0.008). In contrast, whilst there was no significant difference between clusters for anti-Ro52 (K-Wallis, p = 0.059), a direct comparison of C4 (19/63 [30.2%]) with the remaining clusters combined (23/152 [15.1%)) was statistically significant (p = 0.014). There was no significant difference in other autoantibodies or presence of low C3/C4 complement. Concomitant immunosuppressant or corticosteroid use did not differ across clusters.

### Differences in disease activity between clusters

Marked variations in the proportion of patients with active musculoskeletal disease (BILAG A or B score) were found between clusters (p = 0.002); ranging from 19/27 (70.4%) in C1 to 5/24 (20.8%) in C6. Conversely, there was no significant difference between clusters (p = 0.177) in the number of patients with inflammatory arthritis fulfilling the 1997 ACR criterion.

Similarly, BILAG 2004 scores in renal disease was highest in C6 (15/24 [62.5%]) and lowest in C2 (9/36 [25%]) although the proportion of patients with renal disease was not statistically significant between clusters (Chi-2, p = 0.051). The comparison of C6 with the other clusters combined, identified a significant difference (15/23 ([65.2%]) vs. 64/188 [41.2%] respectively, p = 0.005). Although baseline creatinine was significantly different between clusters, it was numerically equal lowest in C6 (60 [52,72] and C3 (60 [53, 70] and highest in C2 (75 [58, 94]) (K-Wallis between clusters p = 0.002).

In terms of ACR criteria, there was also a significant difference in patient numbers that satisfied the renal domain; numerically greatest in C6, (16/24 [66.7%]) and lowest in C2, (9/36 [25%]) (p = 0.017). Oral ulcers were numerically higher in C1 (20/27 [70.1%]) and lower in C5 (14/35 [40%]) (p = 0.036).

### Canonical pathways and network analysis

To investigate relevant protein networks and pathways that underpin each cluster, we identified proteins that were significantly different (higher or lower) in each cluster compared to all other clusters combined, using a linear model adjusted for multiple comparisons. The number of proteins with an adjusted p-value of < 0.05 was: 118 in C1, 67 in C2, 110 in C3, 24 in C4, 49 in C5 and 14 in C6.

We performed pathways analysis using each of the 6 sets of proteins in turn. Pathways with Z score ≥ 1 or Z ≤ − 1 were considered relevant. C1, C2, C3 and C6 each had unique over-represented pathways which were not observed in other clusters (Table 3). The number of these unique pathways varied between clusters and was greatest in C1 (17 unique pathways) and lowest in C6 (one pathway). A total of 18 canonical pathways were common to two or more clusters (Fig. 1C). Notably, the RhoGDI signalling pathway was over-represented in 4 clusters but it was decreased in C2, C4 and C5 and increased in C6.

**Table 3:**
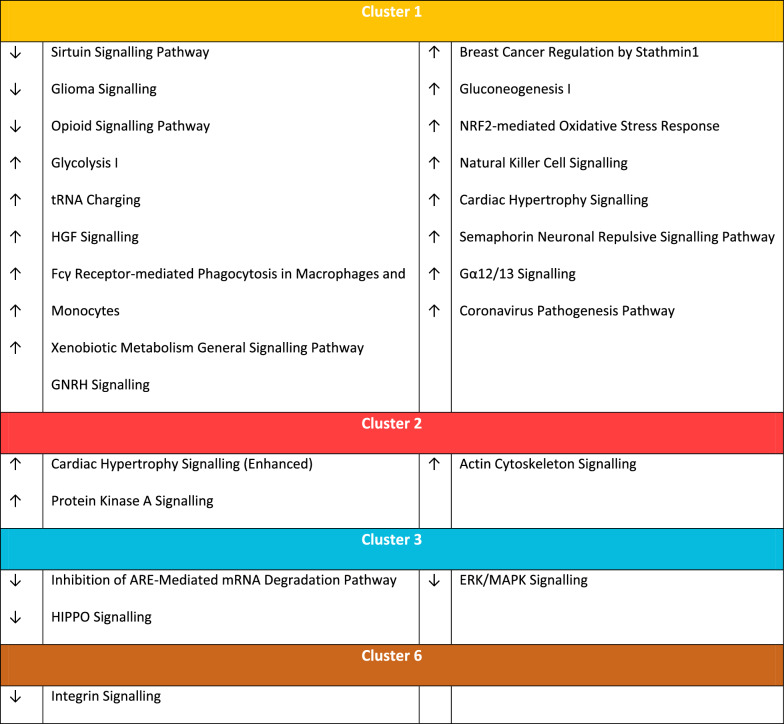
Unique canonical pathways in each cluster

As C1 and C6 were associated with increased frequency of musculoskeletal and renal disease respectively, we performed further analyses on proteins present within these clusters.

### Further stratification of Cluster 1

**Fig. 2:**
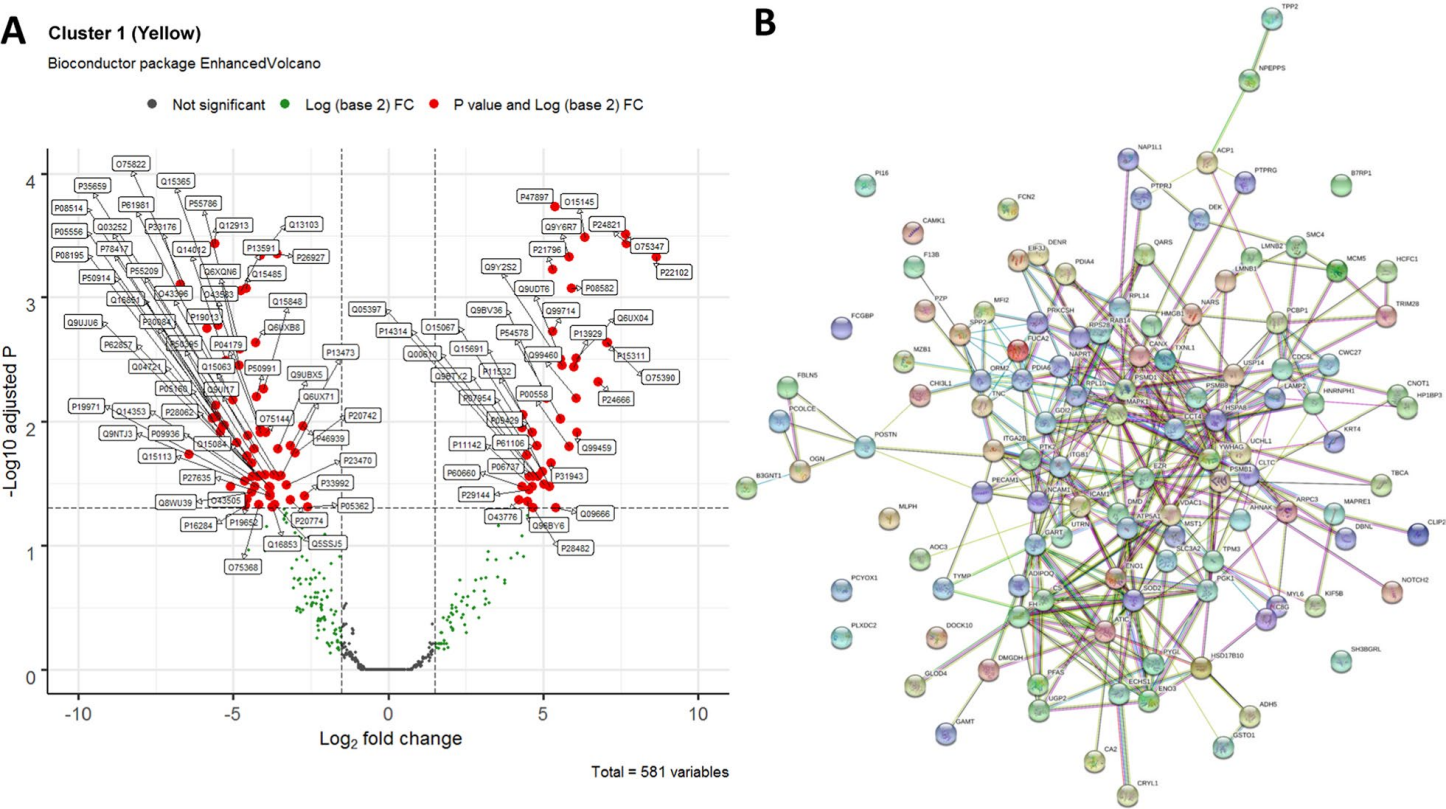
Proteins in C1. **A** Volcano plot showing the individual proteins that are significantly different in C1 compared to the other clusters. The x-axis shows log-fold change and the y-axis − Log10 adjusted P value. Proteins in red are those with a log fold change of < − 1 or > 1 and adjusted P value < 0.05. **B** Network analysis of the proteins significantly different in cluster 1 compared to other clusters

**Fig. 3:**
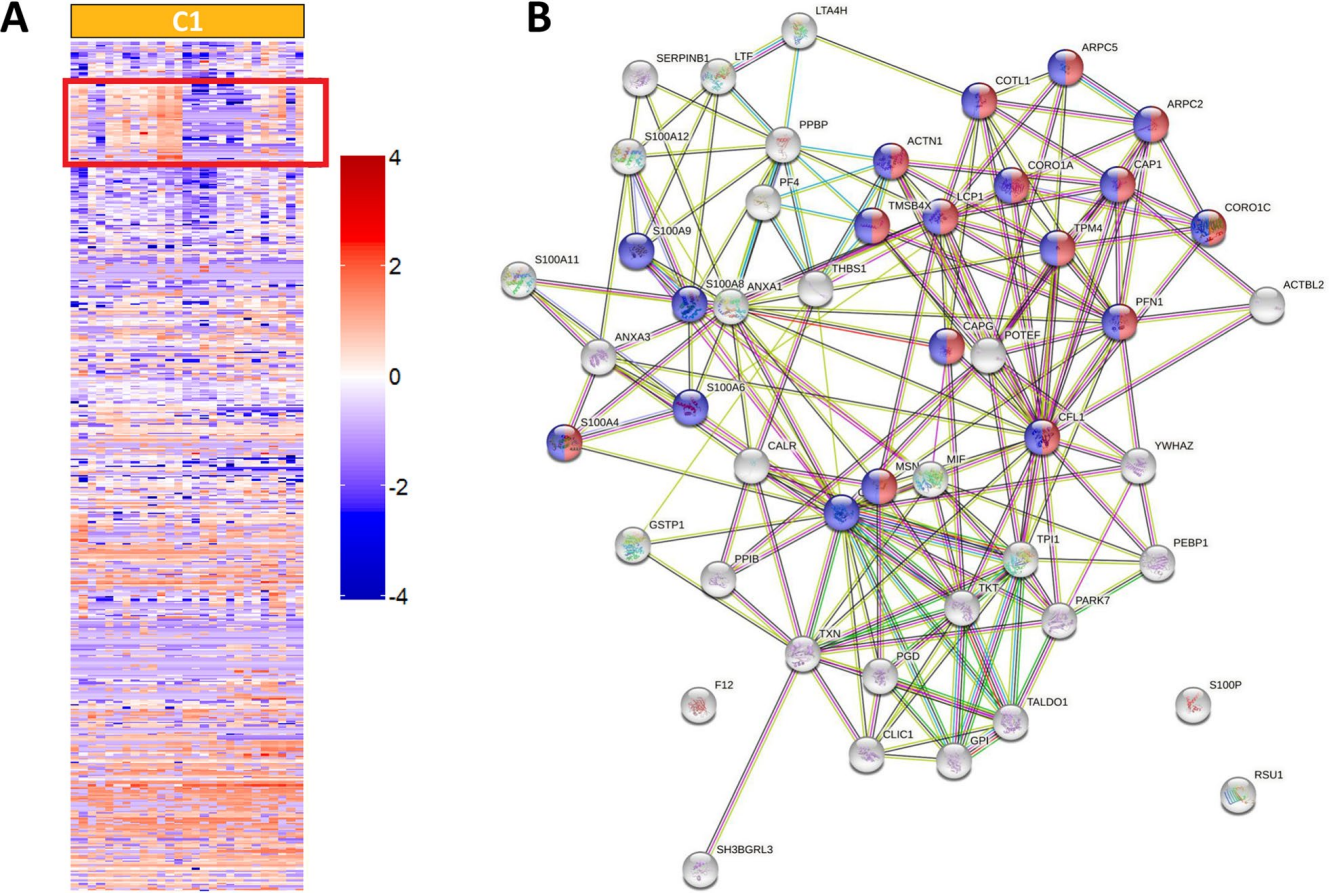
Subcluster analysis of C1A and C1B. **A** Heatmap of proteins significantly different between C1 and the remaining clusters identifies 49 proteins that are different between C1A and C1B. **B** Network analysis of these 49 proteins demonstrating CFL1, LCP1 and CAP1 as central nodes

As MSK disease was over-represented in C1, we aimed to identify which individual proteins were increased/decreased in C1 and whether we could identify key biological pathways. We identified a total of 118 proteins which were significantly different in C1 compared to the other 5 clusters (Fig. 2A), and network analysis of these 118 proteins identified a number of central nodes (Fig. 2B). Furthermore, within the t-SNE plot (Fig. 1A), the C1 cluster appeared to form 2 distinct subclusters which we arbitrarily designated C1A as the cluster closest to C4 (purple) and the other as C1B (Fig. 1B). On evaluation of the heatmap of C1, a subgroup of 49 proteins within C1 was identified as being different between sub-clusters 1A and 1B (Fig. 3A). Of these, 47/49 (96.0%) were numerically higher in C1B, driving this group towards C3 and C6 where they were also increased (the 2 numerically lower proteins were transketolase [TKT] and coagulation factor XII). In GO analysis, the top five pathways which were differentially represented in C1A and C1B were “actin binding”, “cytoskeletal protein binding”, “calcium ion binding” and “Receptor for Advanced Glycation End products (RAGE) receptor binding” and “protein binding”. Network analysis (Fig. 3B) suggested that Cofilin-1 (CFL1), was a central node in the proteins which defined C1B compared to C1A. Seven of the 49 proteins were S100 proteins including S100A8, S100A9, S100A11 and S100A12 (see Additional file 1). After correction for multiple testing (t-test with Holm-Sidak correction) 2 proteins were significantly higher in C1B than C1A: S100A4 and Transaldolase (TALDO1). We found no differences in other disease features, medications, or serology between clusters C1A and C1B (data not shown).

### Association between plasma proteins and renal disease

**Fig. 4:**
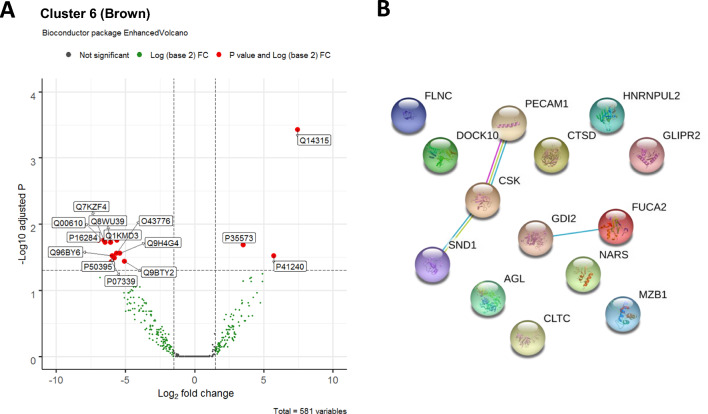
Proteins in C6. **A** Volcano plot showing the individual proteins that are significantly different in C6 compared to the other clusters. Proteins in red are those with a log fold change of < − 1 or > 1 and adjusted P value < 0.05. **B** Network analysis of the proteins significantly different in cluster 1 compared to other clusters

As C6 had over-representation of patients with renal disease, we aimed to identify whether the proteins driving C6 belonged to one or more biological pathways. Of the 14 significantly different proteins in renal disease enriched cluster, C6, 3 were higher and 11 were lower compared to other clusters (Fig. 4A), network analysis did not identify a central node nor a distinct relationship between several nodes (Fig. 4B). Most canonical pathways related to C6 were predicted to be downregulated except for “Rho GDI signalling” and “SLE in T cell signalling pathway”. The only canonical pathway unique to C6 was “Integrin signalling” (for details of the pathway analysis related to C6 see the Additional file 1). Several canonical pathways, including Rac/Rho/Cdc42 pathways, were shared between C2 and C6 but in opposing directions.

We then wanted to know whether any of these 14 proteins were associated with renal disease beyond those patients in C6 after accounting for important confounders. We used an adaptive lasso regression model to select those proteins from C6 which were associated with active renal disease (BILAG A or B) across the whole cohort. In the model, three proteins (Staphylococcal nuclease domain-containing protein 1 [SND1], glycogen debranching enzyme [AGL] and marginal zone B- and B1-cell-specific protein [MZB1]) were selected at lambda 57; higher levels of AGL and lower levels of SND1 and MZB1 were associated with active renal disease. In these models, MZB1 was considered binary (detectable/undetectable) whilst SND1 and AGL were ordinal (high/low/undetectable). Multivariable logistic regression models of active renal disease were constructed with age, gender, ethnicity, low C3 and/or C4 complement and high anti-dsDNA as covariates. The AUC for this model was 0.7346, increasing to 0.7784 when the three proteins were added as covariates (p = 0.0617). Similarly, the AUC for a model of biopsy-proven lupus nephritis increased from 0.8115 to 0.86 (p = 0.0830) (Table 4). Adding the three proteins to a model of proteinuria did not improve the AUC.

**Table 4:** Logistic regression model for active renal disease across the whole cohort

Outcome	Clinical model AUC ROC	3 proteins alone AUC ROC	Clinical model + 3 proteins AUC ROC	p-value (clinical model vs clinical model + proteins)
Active renal disease (BILAG A or B)	0.7346	0.6735	0.7784	0.0617
Biopsy-proven nephritis	0.8115	0.6947	0.8600	0.0830
Proteinuria (ACR > 70)	0.6691	0.6690	0.7208	0.2162

Clinical model comprises: age, gender, ethnicity, high dsDNA, low C3 and/or C4

Proteins: SND1, AGL, MZ

## Discussion

**Fig. 5:**
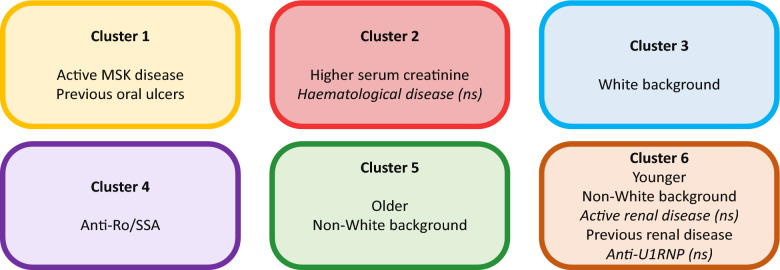
Summary of the 6 proteomic clusters. Key clinical and serological differences in each of the 6 clusters, ns = not statistically significant across all clusters

Using an unbiased approach, we identified 6 proteomic endotypes in a cohort of patients with active SLE (Fig. 5). These clusters were associated with some clinical or serological features, notably inflammatory arthritis, renal disease and anti-Ro/SSA antibodies. It should be noted however, that the tests for statistical significance compare values across all 6 clusters and our reporting of over or under-represented disease features is descriptive based on numerical values. We also identified 3 proteins, (Staphylococcal nuclease domain-containing protein 1 (SND1), glycogen debranching enzyme (AGL) and marginal zone B- and B1-cell-specific protein (MZB1) that were associated with the presence of active renal disease.

In a previous study by Idborg et al. [26], 281 proteins were measured by antibody suspension bead array with the proteins selected based on existing published candidate biomarkers, microarray data and LC–MS data. Using generalised linear models, higher levels of interferon regulating factor 5 (IRF5), solute carrier family 22 member 2 (SLC22A2) and S100 calcium binding protein A12 (S100A12) were identified in SLE patients compared to healthy matched controls; in our data the S100A12 pathway was increased in our C1B subcluster. Using unsupervised clustering, Idborg identified 3 clusters of patients characterised by rheumatoid factor (RF)-IgM, and high or low levels of IRF5; the low IRF5 group closely resembled healthy controls. As might be expected, the RF-high group had increased frequency of anti-Ro/SSA and anti-La/SSB antibodies and a reduced frequency of nephritis. Associations between these molecular subgroups and other clinical features of SLE are not reported.

In our study there was over-representation of active musculoskeletal disease in C1, and a reciprocal under-representation in C6. Interestingly, there was no difference in number of patients fulfilling the 1997 ACR arthritis criterion suggesting that the protein signature is related to active MSK disease, or possibly non-arthritis MSK disease (e.g. myositis). The over-represented pathways in C1 included intracellular proteins associated with regulation of cell cytoskeleton. The P21 activated kinase (PAK) pathway was predicted to be increased in C1 and decreased in C6. Increased PAK signalling has been observed in fibroblast-like synoviocytes (FLS) and associated with joint damage in RA patients [27].

Data visualisation suggested that C1 comprised two distinct sub clusters (1A and 1B) suggesting that 2 molecular subtypes may exist within patients with active MSK disease. The splitting of C1 into these sub-clusters appeared to be due to differences in 49 proteins which have roles in actin binding, cytoskeletal protein binding, calcium ion binding, RAGE receptor binding and protein binding. Cofilin-1 (CFL1) was a central node in the network analysis and has roles in actin and cytoskeletal protein binding. CFL1, which is stimulated by TNFα and GM-CSF, disassembles actin filaments during cellular replication, facilitating FLS migration in RA patients [28]. In a small study by Ooka et al., anti-CFL-1 antibodies were found in 6.3% of patients with SLE but also patients with RA, Behcet’s disease and myositis [29] suggesting that CFL-1 may be an autoantigen common to several inflammatory diseases. Proteins in the S100 family also differed between C1A and C1B. These proteins act as damage-associated molecular patterns (DAMPs) and plasma levels are increased in both adult and childhood-onset SLE, especially those with active disease [30, 31] Whilst increased S100 proteins are associated with lupus nephritis [32], our study suggests that they may also be relevant to patients with lupus arthritis, supporting observations in other forms of inflammatory arthritis [33]. S100A4 was significantly increased in C1B and in RA, S100A4 is expressed in synovium and induces the expression of matrix metalloproteinases [34]. Transaldolase was also increased in cluster C1B. In RA, monocytes have increased expression of Transaldolase which has been proposed to protect RA monocytes from apoptosis, increasing the pool of activated monocytes in inflamed synovium [35].

Cluster 6 had the greatest proportion of renal disease (lowest in C2); these patients were younger and more likely to be non-Caucasian, representing the typical demographic of lupus nephritis [36]. In our data, the integrin signalling pathway was predicted to be reduced in C6, although the role of integrins in SLE remains to be elucidated. Mutations in the Integrin Subunit Alpha M (ITGAM) gene which encodes the CD11b chain of the Mac-1 integrin is a risk factor for SLE [37], the exact mechanisms by which changes in CD11b drives inflammation but has been proposed to be through TLR inhibition of cytokine production [38]. In our pathway analysis, the “integrin signalling” pathway contained the proteins integrin αM, β1 and β3, but not the β2 integrin, which is associated with the development of SLE [39] Although these studies do not explore the clinical variation, one study in 2009 by Yang et al., observed a strong association in lupus nephritis with the mutation, and of note, also observed a higher incidence of arthritis in the absence of the mutation [40]. More research to identify the roles of these integrin subunits in lupus nephritis is required.

Kwon et al. [41], performed MS on urinary samples of SLE patients with and without lupus nephritis compared to HC. They identified an increase in 143 and 67 proteins in patients with SLE without nephritis and with nephritis respectively. They did not perform clustering analysis on their cohort; however, identified 23 common proteins between the 2 SLE groups compared to HC, five of these (ORM1, antithrombin‐III [SERPINC1], ceruloplasmin, haemoglobin subunit beta [HBB] and delta [HBD]) were significantly upregulated in patients with lupus nephritis. We did not detect ORM1 in our data, but the other 4 proteins were not significantly increased in C6 compared to the rest of the cohort, and thus were not included in our lasso model. In a small study, serum Annexin 2 as measured by ELISA, was associated with proliferative lupus nephritis but not membranous disease [42]. In our study, Annexin 2 was detected in fewer than 25% of samples and thus excluded from further analysis. In our data we do not have details of the subtypes of lupus renal disease which limited any subgroup analyses of patients with lupus nephritis.

Only three of the significantly different proteins in C6 had increased levels: filamin-C (FLNC), tyrosine-protein kinase CSK (CSK) and glycogen debranching enzyme (AGL). CSK is associated with autoimmunity as higher levels in early stages of B cell maturation increase the number auto-reactive B cells and autoantibody production [43] and contrary to our study, was significantly downregulated in a pilot proteomic study by Zhou et al. [44], which had a cohort of SLE mostly enriched with cutaneous disease, the observed reduced CSK expression may reflect the differing treatment paradigms of skin versus systemic disease but may also reflect the variation in disease pathogenesis of SLE. Among the proteins with reduced expression, marginal zone B- and B1-cell-specific (MZB1) and platelet endothelial cell adhesion molecule (PECAM-1) are associated with SLE.

PECAM-1 is an important regulator of B cell development and B cell receptor activation. In murine models, PECAM-1 deficiency leads to B cell hyper-responsiveness and autoantibody formation [45]. It has been noted to be elevated in both the urine and serum of patients with SLE [46, 47]. Increased PECAM-1 in the serum of patients with SLE may also be modulated by a greater proportion of metabolic syndrome risk factors including increased age, Body Mass Index (BMI) and waist circumference which may increase PECAM-1 levels. Plasma levels of PECAM-1 were not measured, but leakage of PECAM-1 from plasma into the urine in patients with proteinuria may explain the lower levels that we observed in our study. Further studies with contemporaneous measurement of PECAM-1 in plasma and urine should be considered in patients with lupus nephritis.

MZB1 is implicated in B cell antibody production with elevated levels reported in lymph nodes of lupus patients [48]. MBZ1 is also expressed in plasmocytic dendritic cells and regulates IFNα production, a key cytokine in SLE [49]. In our study, plasma levels of MZB1 were paradoxically lower in C6 although this may again be due to increased excretion via the kidney. To identify whether these biomarkers may have clinical utility, and to control for important confounders such as ethnicity, we developed models of active renal disease using proteins identified from C6. A combination of three proteins improved accuracy of the model to identify patients with active nephritis. Although replication of these findings in an independent cohort is needed, this confirms that data reduction using cluster analysis followed by variable selection is a valid approach for biomarker discovery in patients with active SLE.

The Rac/Rho/Cdc42 pathways, which are associated with cell motility, were differentially modulated in C2 and C6, and the role of these proteins in plasma warrants further investigation. The Rho/Rho kinase pathway is implicated in the pathogenesis of lupus as Rho kinase inhibitors ameliorate SLE disease activity in murine models [50, 51]. The RhoA-Rho kinase pathway is also implicated in B cell activation and survival [52]. If dysregulation of this pathway, reduces B cells activation or survival, this could explain the lower levels of autoantibodies in C2. Between the clusters, there was also a significant difference in number of patients with anti-SSA/Ro antibodies; highest in C4 and lowest in C2. This observation was clearer for Ro60 isotype than Ro52, although this may reflect insufficient power to detect differences between groups. Interestingly, there was no increase in clinical features often associated with anti-SSA/Ro antibodies including mucocutaneous, MSK, neurological or pulmonary disease in C4 compared to other clusters (nor reduced frequency of these features in C2) [53].

A strength of SWATH-MS is the compilation of large spectral libraries containing all known peptide fragments with good reproducibility [12]. Given this, SWATH-MS has been utilised in drug discovery and biomarker identification in gastroenterology, oncology and cardiology [54–57]. SWATH-MS has been used to identify biomarkers in other rheumatic diseases including SS [58] and osteoarthritis [59]. In the study of SS by Cecchettini et al., differentially expressed inflammatory proteins in the saliva of patients with primary SS and HC, were used perform gene ontology analysis which identified some biological processes which were also noted in our study including gluconeogenesis (increased in C1) and protein kinase A signalling (increased in C2) [58].

A strength this study design is the timing of patient plasma collection, which allowed us to capture the proteome profile of high disease activity patients. We found no differences in drug treatment between clusters, suggesting the protein signatures are the result of the disease, rather than drug effect, although longitudinal studies are important to validate these observations.

An important limitation is that only active SLE patients were included in this study, and therefore we cannot determine how the plasma proteome would compare to patients with inactive SLE or HC. As this is also a cross sectional study, it would also be important to perform a longitudinal study with HC to further assess the clinical associations of identified proteins. A study in 2022 by Zhang et al. explored the metabolic profile of 21 HC, 52 SLE patients and 43 LN patients using Ultra high-performance liquid/gas chromatography-tandem mass spectrometry (UPL/GC–MS/MS) [60]. This study discovered 28 differential metabolites, five of which were discriminatory for LN from SLE and significantly associated with urea, creatinine, Cystatin C and C1q, not observed in healthy controls and reiterating the importance of a control group for validation.

Furthermore, as most patients recruited into BILAG-BR, are patients with refractory disease requiring biological therapy, patients with early or naïve disease are not represented in this study, noting that the activity scores used to stratify patients are for purposes of disease monitoring and making treatment decisions, but may not reflect disease biology. The lack of significant differences in SDI and disease duration between clusters at least implies that the patients are relatively homogeneous in terms active and severe disease.

As each of the 6 clusters were relatively small, this study may lack power to identify differences in some clinical or serological features. Similarly, as the most frequent active organ domains were mucocutaneous, MSK and renal, we may lack power to identify differences in other domains such as neuropsychiatric disease. Importantly, SWATH-MS is not suited to detecting low level proteins such as cytokines and chemokines and future studies including multiplex cytokine/chemokine data should be integrated into proteomic analysis. Interestingly, in our data there was a lower-than-expected proportion of patients taking AM, which may reflect the refractory stage of disease in many patients within the BILAG-BR or incomplete data capture; the proportion of ever used AM was more than 90%. Importantly, we saw no difference in AM use between clusters considering ‘current’, or ‘ever use’ was considered.

## Conclusion

In conclusion, SWATH-MS is a valid method for identifying proteomic differences in patients with SLE and can identify proteins which may be useful biomarkers for features of active disease, notably MSK and renal involvement. The pathways and proteins identified may serve as potential biomarkers and/or therapeutic targets and investigation of their role in SLE pathogenesis is warranted.

## Supplementary Information

**Additional file 1: Figure S1.** ‘Elbow’ Methods of determining optimal cluster number with the RStudio package ‘nbclust’. **Table S1.** Detailed ethnicity data for the study population. **Table S2.** Details of ethnicity between clusters. **Table S3.** List of Proteins in Cluster 1 with Significant FC and P value. **Table S4.** Cluster 1 Proteins associated with subcluster 1A and 1B. **Figure S2.** Dissimilarity matrices for each of the 6 clusters. **Table S5.** Proteins and canonical pathways relating to the clusters 1A and 1B. **Figure S3.** Gene Ontology of all proteins in cluster 1 utilising CLUEgo, a Cytoscape plugin for biological interpretation (4). **Table S6.** Pathway analysis of the proteins which are increased or decreased in C6 compared to the rest of the cohort.

## Data Availability

The datasets used during the current study may be available from the corresponding author on reasonable request providing anonymisation can be maintained.

## Abbreviations

ACR: American College of Rheumatology
AGL: Glycogen debranching enzyme
AM: Anti-malarial
BEL: Belimumab
BILAG-BR: British Isles Lupus Assessment Group-Biologics Registry
BMI: Body Mass Index
C1: Cluster 1
C2: Cluster 2
C3: Cluster 3
C4: Cluster 4
C5: Cluster 5
C6: Cluster 6
CFL-1: Cofilin1
CSK: Tyrosine-protein kinase
DAMPs: Damage-associated molecular patterns
DDA: Data-dependant acquisition
FLNC: Filamin-C
FLS: Fibroblast-like synoviocytes
HC: Healthy Control
IRF5: Interferon regulating factor 5 (IRF5)
ITGAM: Integrin subunit alpha M
LC-MS: Liquid chromatography mass spectrometry
MALDI-TOF MS: Matrix assisted laser desorption ionization-time of flight mass spectrometry
MMF: Mycophenolate Mofetil
MS: Mass-spectrometry
MZB1: Marginal zone B- and B1-cell-specific protein
NICE: National Institute for Health and Care Excellence
PAK: P21-activated kinases
PECAM-1: Platelet endothelial cell adhesion molecule
PLC: Phospholipase C
RA: Rheumatoid arthritis
RAGE: Receptor for advanced glycation end products
RF: Rheumatoid factor
RTX: Rituximab
S100A12: S100 calcium binding protein A12
SS: Sjogren’s Syndrome
SLC22A2: Solute carrier family 22 member 2
SLE: Systemic lupus erythematosus
SND1: Staphylococcal nuclease domain-containing protein 1
SWATH-MS: Sequential window acquisition of all theoretical mass spectra mass spectrometry
TA: Targeted acquisition
t-SNE: T-distributed stochastic neighbour embedding
uACR: Albumin-creatinine ratio
uPCR: Urine protein-creatinine ratio
