# Supplementary material for: Proteomic analysis identifies subgroups of patients with active systemic lupus erythematosus
Source: Clin Proteomics. 2023 Jul 29;20:29. doi: 10.1186/s12014-023-09420-1 (PMC10385905; doi:10.1186/s12014-023-09420-1)
Supplement: Supplementary file 1 — Additional file 1: Figure S1. ‘Elbow’ Methods of determining optimal cluster number with the RStudio package ‘nbclust’. Table S1. Detailed ethnicity data for the study population. Table S2. Details of ethnicity between clusters. Table S3. List of Proteins in Cluster 1 with Significant FC and P value. Table S4. Cluster 1 Proteins associated with subcluster 1A and 1B. Figure S2. Dissimilarity matrices for each of the 6 clusters. Table S5. Proteins and canonical pathways relating to the clusters 1A and 1B. Figure S3. Gene Ontology of all proteins in cluster 1 utilising CLUEgo, a Cytoscape plugin for biological interpretation (4). Table S6. Pathway analysis of the proteins which are increased or decreased in C6 compared to the rest of the cohort. [file 12014_2023_9420_MOESM1_ESM.docx]

**Proteomic analysis identifies subgroups of patients with active systemic lupus erythematosus**

**SUPPLEMENTARY FILE**

**SWATH-MS Methods and Calibration**

# **Sample Preparation**

Plasma starting material (10 µL), for each sample, from study wide pool, or commercially supplied plasma (BioIVT, US) was depleted of albumin, IgG, transferrin, fibrinogen, IgA, α-2-macroglobulin, α-1-antitrypsin, IgM, haptoglobin, α1-acid glycoprotein, apolipoprotein A-I and apolipoprotein A-II, which constitute 95% total protein (Top-12 Depletion spin columns, Thermo Scientific UK). Depleted plasma was buffer exchanged and concentrated by centrifugal filtration (Amicon ultra 0.5 3kDa molecular weight cut-off, Sigma-Aldrich UK) into digestion buffer (25 mM ammonium bicarbonate) yielding a final volume of 80-100 µL . Total protein concentration was determined by microplate format BCA Assay (BCA Protein Assay Kit, Thermo Scientific UK; SpectraMaxi3x, Molecular Devices US). Each sample (96 µL total volume, 50 µg total protein, 25 mM ammonium bicarbonate) was reduced and solubilised by addition of dithiothreitol (12 µL, 50 mM, 25 mM ammonium bicarbonate) and sodium deoxycholate (12 µL, 10%, 25 mM ammonium bicarbonate). The reduction mixture (120 µL, 0.33 µg µL^‑1^protein, 25 mM ammonium bicarbonate, 5 mM dithiothreitol, 1% sodium deoxycholate) was incubated at 60 °C for 30 min with vortex mixing at 300 rpm. Reduced protein was alkylated by addition of iodacetamide (10 µL, 650 mM, 25 mM ammonium bicarbonate) and alkylation mixture (130 µL, 0.30 µg µL^‑1^protein, 50 mM iodacetamide, 25 mM ammonium bicarbonate, 0.9% sodium deoxycholate) was incubated at 24 °C in the dark for 30 min. Reduced and alkylated protein was digested by addition of trypsin (10 µL, 0.1 µg µL^‑1^, 25mM ammonium bicarbonate) to give protein:enzyme ratio of 50:1. Digest mixture (140 µL, 0.28 µg µL^‑1^protein, 25 mM ammonium bicarbonate, 0.9% sodium deoxycholate) was incubated at 37 °C overnight with vortex mixing at 300 rpm. Digested protein mixture was acidified to pH < 4.0 by addition of formic acid (140 µL, 1%). Sample was recovered from deoxycholate precipitate by centrifugation at 12,000×g for 10 mins at 10°C. Peptides were lyophilised from the recovered supernatant by vacuum centrifugation (MiVac Quattro Concentrator, SP Scientific US) and stored at ‑80 °C until required.

# **LC-MS Instrument Configuration**

Samples were analysed by SWATH-MS with a micro-flow LC-MS system comprising an Eksigent nanoLC 400 autosampler and an Eksigent nanoLC 425 pump coupled to a Sciex 6600 Triple-TOF mass spectrometer with a DuoSpray Ion Source. System was configured for a trap-elute elute analysis in which sample was injected from the autosampler (8°C) onto a trap column (YMC- Triart C18; length: 5 mm; ID: 0.5 mm; particle size: 3 µm; pore size: 120 Å) with loading buffer mobile phase (10 µL min^-1^, 3 min, 2% acetonitrile, 0.1% formic acid) then eluted through an analytical column (YMC-Triart C18; length: 150 mm; ID: 0.3 mm; particle size: 3 µm; pore size: 120 Å; 30°C) with the required analytical gradient into the mass spectrometer source. The system was controlled by Analyst software v1.7.1 and eksigent control software v4.2.

# **Instrument Suitability Verification**

Prior to data acquisition the mass spectrometer was tuned and calibrated according to manufacturer’s instructions and the performance of the LC-MS system was verified as follows. Sensitivity, mass accuracy and resolution of the 6600 mass spectrometer was verified by infusion of Sciex tuning solution (5 µL min^-1^, 5500 V) against the following specification: In high resolution TOFMS scan type signal intensities of ion at m/z 132 greater than 5e5 cps and m/z 829 2e5 cps and 32,000 resolution; in high resolution product ion scan of m/z 829 at collision energy 10 eV intensity of m/z 829 ion equal to or greater than signal in TOFMS scan; in high resolution product ion scan of m/z 829 at collision energy 45 eV intensity of m/z 494 ion greater than 2.3e4 cps; in high sensitivity product ion scan of m/z 829 at collision energy 45 eV intensity of m/z 494 ion greater than 4.7e4 cps and 15,000 resolution. Performance of the LC system was verified by injection of an artificial peptide mixture (4 µL, 10 fmol µL^‑1^, pepCalMix - Sciex, 2% acetonitrile, 0.1% formic acid) with analytical gradient (5 µL min^-1^, 5 min, 3-35% acetonitrile, 0.1% formic acid) and mass spectrometer method alternating TOFMS (m/z 405-1250) and product scan (product of 758.91, collision energy 40 eV, m/z 100-1500) and checking the results against the following specification: Overlay of XIC chromatograms for each of the component peptides ion with width m/z 0.2 in the peptide mixture show Gaussian peaks with FWHM less than 5 s distributed over retention time 4-7.5 min and dominant ion m/z 964.977 with retention time close to 6.5 min with intensity greater than 1e6 cps whilst in the TIC of product ion scan of m/z 758.91 a single dominant peak at retention time 6-6.5 min with maximum intensity greater than 2e5 and XIC of daughter ion m/z 1070.59 width m/z 0.05 between 8000 and 16000 cps. LC-MS system suitability was further verified by injection standard human peptide (2.5 µL, 0.8 µg µL^‑1^, MS Compatible Human Protein Extract, Digest - Promega, 0.2 IE µL^‑1^ iRT - Biognosis Switzerland, 2% acetonitrile, 0.1% formic acid) with analytical gradient (5 µL min^-1^, 5 min, 8-35% acetonitrile, 0.1% formic acid) and DDA mass spectrometry method (top 75 ions in m/z rage 400-1250 with charge 2-4 and intensity over 1000 cps excluded for 10 s after one occurrence) verifying that over 60% of MSMS spectra obtained were allocated a quality score greater than 10 by Peakview software v2.1 in IDAexplorer mode.

# **Sample Data Acquisition and Continuous Quality Control**

Lyophilised peptide samples were reconstituted in loading buffer (62.5 µL, 2% acetonitrile, 0.1% formic acid, 10 fmol µL^‑1^ PepCalMix Sciex UK, 2 IE iRT peptides from Biognosis Switzerland) and loaded onto the autosampler in batches of 8-24 for sequential SWATH-MS analysis. In addition to samples injection sequences included QC standards injected for SWATH-MS analysis as per the samples, a blank injection of loading buffer after each SWATH-MS and a auto-calibration injection of pepCalMix every eighth injection (4 µL, 10 fmol µL^‑1^, pepCalMix - Sciex, 2% acetonitrile, 0.1% formic acid) both using the pepCalMix method described for instrument verification above. Typically, injection sequences began with standard human peptide (8 µL, 0.8 µg µL^‑1^, MS Compatible Human Protein Extract, Digest - Promega, 0.2 IE µL^‑1^ iRT - Biognosis Switzerland, 2% acetonitrile, 0.1% formic acid) followed by local standard commercial plasma digest (8 µL, 0.8 µg µL^‑1^, 0.2 IE µL^‑1^ iRT - Biognosis Switzerland, 2% acetonitrile, 0.1% formic acid). For the rest of the injection sequence after every fourth sample injection a quality control was acquired, this alternated between the standard human peptide as above, and either a digest of a pool of all sample in the study (8 µL, 0.8 µg µL^‑1^, 0.2 IE µL^‑1^ iRT - Biognosis Switzerland, 2% acetonitrile, 0.1% formic acid) or the commercial plasma digest as above. Throughout the batch human peptide analysis were monitored to ensure that: The total TIC remained 1-2e8 and did not drop by more than 20% during the batch; MS^1^ TIC similar in intensity to total TIC; MS^1^ TIC and sum off MS^2^ TICs retained an approximate Gaussian profile. Pooled study and commercial plasma digest data was used to monitor instrument performance across the facility over time. For MS-SWATH analysis itself samples were eluted with an analytical gradient (5 µL min^-1^, 103 min, 3-40% acetonitrile, 0.1% formic acid) and mass spectrometer method with a total duty cycle of 2.8 s comprising a TOF MS^1^ scan (m/z 400-1250, 250 ms) followed by 100 SWATH-MS scans (m/z 100-1500, 25ms) with variable m/z isolation widths, collision energy and collision energy spread.

**Data alignment**

SWATH maps were searched with the OpenSWATH algorithm (version 2.0.1) against a spectral library consisting of plasma proteins. This spectral library was generated on the basis of a previously published plasma protein library (1) but updated to be compatible with a 100 variable window acquisition method employed in the Stoller Centre (See supplementary methods). OpenSWATH results of each SWATH map were subsequently subjected to the pyProphet algorithm (version 0.18.3) which assesses each inferred transition and assigns a statistical FDR value (necessary for filtering during alignment).

OpenSWATH maps were aligned with the TRIC (msproteomicstools version 0.4.3) feature alignment algorithm (2) with the following parameters:

-method LocalMST

-realign_method lowess

-max_rt_diff 60

-mst:useRTCorrection True

-mst:Stdev_multiplier 3.0

-target_fdr 0.01

-max_fdr_quality 0.05

The aligned SWATH maps are processed with MSstats to infer protein-level quantification based on the aligned transition-level quantitative information. Protein quantification was performed with “top3” option for parameter “featureSubset” and normalisation with Tukey-Median Polish (TMP).

Samples were subjected to coefficient of variation calculations to retain high quality samples. Coefficient of variation calculations were performed across duplicates where samples that met a 20% and 30% CV value at the median and 75^th^ percentile respectively were chosen for further downstream analysis.

Proteins that were present in low quantities but below the threshold of detection within the plasma was designated being absent with this technique.

**Clustering algorithms**

The number of clusters was determined using the package *nbclust* for R. Each algorithm estimates the number of clusters. For further analysis the mode value was used.

**R studio package *nbclust* methods of cluster validation utilised (3)**

1. Krzanowski and Lai
2. Calinski and Harabasz
3. Hartigan
4. Cubic Clustering Criterion
5. nlog([T]|[W])
6. k^2^|W
7. TraceCovW
8. TraceW
9. TraceW^-1B^
10. [T]/[W]
11. C-Index
12. Davies and Bouldin
13. Silhouette
14. Je(2)/Je(1)
15. P seudot^2^
16. Beale
17. ĉ/k^.5^
18. Ball and Hall
19. Point-Biserial
20. Gap
21. Frey and Groenewood
22. McClain and Rao
23. Gamma
24. G(+)
25. Tau
26. Dunn
27. Modified statistic of Hubert
28. SD
29. Lebart
30. SDbw

**Supplementary Data Figures and Tables**

**Figure S1: ‘Elbow’ Methods of determining optimal cluster number with the RStudio package ‘nbclust’.**


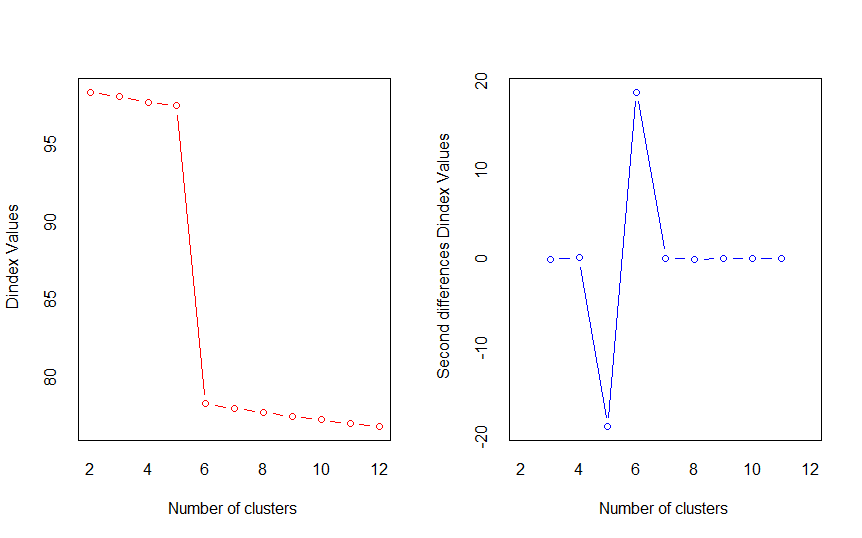


**Table S1. Detailed ethnicity data for the study population**

| **Ethnicity** | **Total (%)** |
| --- | --- |
| **African** | 9 (4.0) |
| **South East Asian** | 42 (18.8) |
| **Caribbean** | 10 (4.5) |
| **Missing** | 1 (0.004) |
| **Not specified** | 13 (5.8) |
| **Other Black** | 3 (1.3) |
| **Other Ethnic** | 4 (1.8) |
| **Other Mixed** | 2 (0.9) |
| **Caucasian** | 132 (59.1) |
| **White and Black African** | 2 (0.9) |
| **White and Black Caribbean** | 5 (2.2) |

*Note: Caucasian: Sum total of White British, White Irish and Other White. South East Asian: Sum total of Bangladeshi, Chinese, Indian, Other Asian and Pakistani.*

**Table S2. Details of ethnicity between clusters**

| **Ethnicity** | **Cluster 1** | **Cluster 2** | **Cluster 3** | **Cluster 4** | **Cluster 5** | **Cluster 6** |
| --- | --- | --- | --- | --- | --- | --- |
| African | 0 | 1 | 1 | 4 | 2 | 1 |
| South East Asian | 4 | 6 | 5 | 11 | 9 | 8 |
| Caribbean | 2 | 1 | 0 | 0 | 3 | 4 |
| Missing | 0 | 0 | 0 | 1 | 0 | 0 |
| Not Specified | 1 | 2 | 2 | 2 | 3 | 3 |
| Other Black | 1 | 1 | 0 | 0 | 1 | 0 |
| Other ethnic | 0 | 0 | 0 | 1 | 2 | 1 |
| Other Mixed | 0 | 0 | 0 | 1 | 1 | 0 |
| Caucasian | 17 | 25 | 26 | 43 | 14 | 7 |
| White & Black African | 0 | 0 | 0 | 1 | 1 | 0 |
| White & Black Caribbean | 2 | 0 | 2 | 1 | 0 | 0 |

*Note: Caucasian: Sum total of White British, White Irish and Other White. South East Asian: Sum total of Bangladeshi, Chinese, Indian, Other Asian and Pakistani.*

**Table S3: List of Proteins in Cluster 1 with Significant FC and P value**

| **Onevsall** | **logFC** | **AveExpr** | **t** | **P.Value** | **adj.P.Val** |
| --- | --- | --- | --- | --- | --- |
| A5YKK6 | 9.232636 | 5.370888 | 6.630531 | 1.25E-10 | 7.26E-08 |
| Q9UHG3 | -4.00296 | 9.370003 | -6.45118 | 3.42E-10 | 9.93E-08 |
| Q13263 | 10.12026 | 4.546931 | 6.309242 | 7.48E-10 | 1.45E-07 |
| P20618 | 9.256143 | 6.029368 | 6.215366 | 1.25E-09 | 1.81E-07 |
| P31939 | 8.105135 | 5.422695 | 5.927787 | 5.8E-09 | 6.74E-07 |
| P07360 | 5.655766 | 9.392598 | 5.859968 | 8.27E-09 | 8.01E-07 |
| P00918 | -4.10043 | 9.405052 | -5.82745 | 9.8E-09 | 8.13E-07 |
| P27824 | 8.887578 | 5.193795 | 5.784979 | 1.22E-08 | 8.87E-07 |
| P36222 | -7.45577 | 7.853506 | -5.36982 | 9.9E-08 | 6.39E-06 |
| P13667 | 6.984288 | 4.949442 | 5.161939 | 2.71E-07 | 1.46E-05 |
| P51610 | 8.481668 | 6.104371 | 5.157907 | 2.76E-07 | 1.46E-05 |
| P11766 | 9.411527 | 8.091496 | 5.05102 | 4.58E-07 | 2.22E-05 |
| P06733 | 7.137863 | 4.070246 | 4.975615 | 6.51E-07 | 2.91E-05 |
| P20700 | 5.590765 | 10.51731 | 4.941614 | 7.62E-07 | 3.16E-05 |
| P06753 | 8.002705 | 5.04954 | 4.914861 | 8.62E-07 | 3.34E-05 |
| P25705 | -5.94998 | 8.296737 | -4.74638 | 1.85E-06 | 6.73E-05 |
| Q9HC38 | -6.76635 | 8.228664 | -4.71463 | 2.14E-06 | 7.3E-05 |
| P47897 | 5.385593 | 3.804219 | 4.487846 | 5.77E-06 | 0.000186 |
| O75347 | 7.658333 | 9.079331 | 4.357913 | 1.01E-05 | 0.000307 |
| O15145 | 6.347208 | 4.423417 | 4.330135 | 1.13E-05 | 0.000328 |
| Q12913 | -5.59828 | 10.61962 | -4.2804 | 1.39E-05 | 0.00037 |
| P24821 | 7.687698 | 5.343504 | 4.278395 | 1.4E-05 | 0.00037 |
| P26927 | -3.59732 | 8.307108 | -4.22353 | 1.75E-05 | 0.000443 |
| Q13103 | -4.12353 | 8.748633 | -4.20498 | 1.89E-05 | 0.000458 |
| Q9Y6R7 | 5.837827 | 4.41955 | 4.182983 | 2.07E-05 | 0.000472 |
| P22102 | 8.642474 | 7.803067 | 4.180099 | 2.11E-05 | 0.000472 |
| P21796 | 5.310296 | 3.504989 | 4.112458 | 2.76E-05 | 0.000593 |
| P33176 | -6.72135 | 6.944204 | -4.032 | 3.81E-05 | 0.00079 |
| Q15485 | -4.57472 | 6.994477 | -4.00067 | 4.3E-05 | 0.000842 |
| P08582 | 5.917928 | 4.233612 | 3.998019 | 4.35E-05 | 0.000842 |
| P13591 | -4.7468 | 5.594773 | -3.97799 | 4.7E-05 | 0.000881 |
| O75822 | -6.44969 | 6.560436 | -3.9547 | 5.16E-05 | 0.000937 |
| O43583 | -5.48246 | 7.774219 | -3.79579 | 9.5E-05 | 0.001672 |
| P19013 | -5.85221 | 5.913997 | -3.77012 | 0.000105 | 0.001789 |
| Q99714 | 5.317129 | 5.506783 | 3.749281 | 0.000113 | 0.001878 |
| P15311 | 7.047946 | 6.828169 | 3.681265 | 0.000147 | 0.002333 |
| Q6UXB8 | -4.27458 | 7.990097 | -3.675 | 0.000149 | 0.002333 |
| O75390 | 7.077044 | 6.227991 | 3.670496 | 0.000153 | 0.002333 |
| P55786 | -4.78393 | 8.417761 | -3.63188 | 0.000175 | 0.002602 |
| Q6UX04 | 6.057252 | 5.451677 | 3.576105 | 0.000215 | 0.003116 |
| Q9UDT6 | 5.562797 | 4.238455 | 3.566822 | 0.000222 | 0.003139 |
| Q14012 | -5.23089 | 8.209022 | -3.54956 | 0.000236 | 0.003259 |
| Q9Y2S2 | 5.619789 | 6.246009 | 3.521798 | 0.00026 | 0.003513 |
| Q6XQN6 | -4.79962 | 4.218499 | -3.51546 | 0.000266 | 0.003513 |
| P13929 | 5.979841 | 4.910724 | 3.498981 | 0.000283 | 0.003653 |
| P24666 | 6.790134 | 7.280042 | 3.417479 | 0.000379 | 0.004791 |
| O43396 | -5.32044 | 5.114543 | -3.3833 | 0.000424 | 0.005239 |
| Q15848 | -4.01157 | 8.397987 | -3.36382 | 0.000453 | 0.005478 |
| P50991 | -4.24505 | 14.318 | -3.31362 | 0.000538 | 0.006374 |
| Q99460 | 6.056472 | 8.431602 | 3.301929 | 0.000563 | 0.006485 |
| Q9BV36 | 5.093196 | 3.744978 | 3.297192 | 0.000569 | 0.006485 |
| P61981 | -5.02072 | 7.147183 | -3.2807 | 0.000602 | 0.006725 |
| P50395 | -5.55605 | 6.748918 | -3.24509 | 0.00068 | 0.007456 |
| P11532 | 4.332318 | 3.878156 | 3.186113 | 0.000825 | 0.008874 |
| Q16851 | -5.54836 | 7.127361 | -3.17229 | 0.000867 | 0.009159 |
| P08195 | -5.68109 | 6.637447 | -3.15838 | 0.000909 | 0.009426 |
| P54578 | 5.558623 | 8.050117 | 3.148365 | 0.000938 | 0.009564 |
| P08514 | -5.2844 | 5.099177 | -3.10951 | 0.001064 | 0.010654 |
| P46939 | -2.76088 | 14.46845 | -3.09252 | 0.001119 | 0.010876 |
| P05556 | -5.33264 | 4.686983 | -3.09282 | 0.001123 | 0.010876 |
| O15067 | 4.315992 | 4.047192 | 3.076541 | 0.001179 | 0.011231 |
| Q15365 | -4.12682 | 4.422991 | -3.0686 | 0.001209 | 0.011333 |
| P05160 | -5.41492 | 11.53761 | -3.04657 | 0.001305 | 0.012032 |
| Q99459 | 6.092383 | 12.45364 | 3.035111 | 0.001363 | 0.012242 |
| P04179 | -4.11694 | 4.332233 | -3.02414 | 0.001394 | 0.012242 |
| Q15691 | 4.642152 | 4.253185 | 3.021945 | 0.001405 | 0.012242 |
| O75144 | -3.93938 | 5.468512 | -3.02004 | 0.001412 | 0.012242 |
| P35659 | -4.52045 | 4.460432 | -2.99645 | 0.001523 | 0.013012 |
| P30084 | -4.87201 | 4.697391 | -2.95087 | 0.00176 | 0.01482 |
| P09429 | 4.802868 | 6.015807 | 2.927191 | 0.001894 | 0.015602 |
| P13473 | -3.14873 | 7.100808 | -2.92399 | 0.001907 | 0.015602 |
| P00558 | 5.849229 | 9.047941 | 2.918064 | 0.001968 | 0.015883 |
| Q9UBX5 | -3.56793 | 6.572449 | -2.8931 | 0.002097 | 0.016684 |
| Q9UI17 | -4.33206 | 8.691971 | -2.88924 | 0.002125 | 0.016684 |
| P20742 | -3.01971 | 14.60659 | -2.86286 | 0.0023 | 0.017815 |
| Q9NTJ3 | -6.4267 | 9.950009 | -2.85839 | 0.002392 | 0.018286 |
| Q9BTY2 | 4.461279 | 4.998924 | 2.839809 | 0.002472 | 0.018653 |
| P78417 | -4.55115 | 5.367987 | -2.83129 | 0.002538 | 0.018905 |
| P50914 | -4.40102 | 3.86816 | -2.78064 | 0.002953 | 0.021547 |
| P31943 | 5.254238 | 7.000531 | 2.781598 | 0.002967 | 0.021547 |
| P07954 | 4.983648 | 11.93158 | 2.721391 | 0.003538 | 0.025378 |
| Q03252 | -3.97442 | 11.94202 | -2.69393 | 0.003805 | 0.026845 |
| P55209 | -3.69235 | 4.38142 | -2.69098 | 0.003835 | 0.026845 |
| Q04721 | -4.17301 | 5.467038 | -2.68049 | 0.00396 | 0.027188 |
| Q6UX71 | -3.46437 | 3.752841 | -2.67825 | 0.003978 | 0.027188 |
| P62857 | -4.17879 | 4.00151 | -2.6571 | 0.004238 | 0.027586 |
| P06737 | 4.544855 | 10.57471 | 2.657199 | 0.004248 | 0.027586 |
| Q00610 | 4.834666 | 8.860633 | 2.657196 | 0.004262 | 0.027586 |
| Q05397 | 4.669436 | 6.432162 | 2.655863 | 0.00427 | 0.027586 |
| Q15063 | -3.53633 | 4.185831 | -2.64957 | 0.004321 | 0.027586 |
| P28062 | -4.22358 | 4.855687 | -2.65047 | 0.004321 | 0.027586 |
| Q14353 | -4.38135 | 5.501825 | -2.64425 | 0.004404 | 0.027811 |
| Q15113 | -4.62543 | 7.080675 | -2.61087 | 0.004858 | 0.030348 |
| P14314 | 5.021357 | 5.591559 | 2.591955 | 0.005156 | 0.031869 |
| P23470 | -3.27859 | 5.071638 | -2.5742 | 0.005349 | 0.032715 |
| O43505 | -5.08812 | 8.651331 | -2.56605 | 0.005558 | 0.033355 |
| P61106 | 5.209128 | 8.863656 | 2.565975 | 0.005572 | 0.033355 |
| P60660 | 4.330817 | 5.069656 | 2.557977 | 0.005626 | 0.033355 |
| P11142 | 4.637187 | 10.85883 | 2.547977 | 0.005807 | 0.033447 |
| P19971 | -4.31157 | 10.80261 | -2.54655 | 0.005808 | 0.033447 |
| Q15084 | -3.85605 | 3.806822 | -2.54497 | 0.005814 | 0.033447 |
| Q9UJU6 | -3.82059 | 4.131652 | -2.53704 | 0.005943 | 0.033853 |
| P29144 | 4.535193 | 8.789994 | 2.516947 | 0.006326 | 0.035681 |
| Q8WU39 | -4.40659 | 8.112788 | -2.49987 | 0.006622 | 0.036993 |
| P09936 | -3.81999 | 3.357477 | -2.47788 | 0.006997 | 0.038716 |
| P33992 | -2.69823 | 2.777731 | -2.46217 | 0.007285 | 0.03993 |
| P27635 | -3.80728 | 3.346307 | -2.45961 | 0.007354 | 0.039931 |
| P19652 | -4.55187 | 14.71454 | -2.43768 | 0.007873 | 0.042355 |
| O43776 | 4.22032 | 5.613366 | 2.428409 | 0.008035 | 0.042829 |
| P20774 | -3.16511 | 3.141258 | -2.41967 | 0.008172 | 0.043161 |
| Q96BY6 | 4.487554 | 5.309687 | 2.413947 | 0.008389 | 0.04391 |
| O75368 | -4.17409 | 4.12737 | -2.3855 | 0.009014 | 0.046754 |
| Q5SSJ5 | -3.6798 | 3.23426 | -2.38034 | 0.009093 | 0.046754 |
| P16284 | -4.61769 | 10.54153 | -2.37438 | 0.009357 | 0.047686 |
| Q16853 | -3.74019 | 5.857194 | -2.35841 | 0.009641 | 0.048709 |
| P05362 | -2.60143 | 8.10957 | -2.34958 | 0.009835 | 0.049261 |
| Q09666 | 5.396903 | 10.34781 | 2.358805 | 0.009957 | 0.049447 |
| P28482 | 4.674558 | 7.012945 | 2.346814 | 0.010086 | 0.049662 |

**Table S4. Cluster 1 Proteins associated with subcluster 1A and 1B**

| **Uniprot ID** | **Protein Name** |
| --- | --- |
| P04406 | Glyceraldehyde-3-phosphate dehydrogenase (GAPDH) (EC 1.2.1.12) (Peptidyl-cysteine S-nitrosylase GAPDH) (EC 2.6.99.-) |
| P02775 | Platelet basic protein (PBP) (C-X-C motif chemokine 7) (Leukocyte-derived growth factor) (LDGF) (Macrophage-derived growth factor) (MDGF) (Small-inducible cytokine B7) [Cleaved into: Connective tissue-activating peptide III (CTAP-III) (LA-PF4) (Low-affinity platelet factor IV); TC-2; Connective tissue-activating peptide III(1-81) (CTAP-III(1-81)); Beta-thromboglobulin (Beta-TG); Neutrophil-activating peptide 2(74) (NAP-2(74)); Neutrophil-activating peptide 2(73) (NAP-2(73)); Neutrophil-activating peptide 2 (NAP-2); TC-1; Neutrophil-activating peptide 2(1-66) (NAP-2(1-66)); Neutrophil-activating peptide 2(1-63) (NAP-2(1-63))] |
| P07996 | Thrombospondin-1 (Glycoprotein G) |
| P02776 | Platelet factor 4 (PF-4) (C-X-C motif chemokine 4) (Iroplact) (Oncostatin-A) [Cleaved into: Platelet factor 4, short form (Endothelial cell growth inhibitor)] |
| P63104 | 14-3-3 protein zeta/delta (Protein kinase C inhibitor protein 1) (KCIP-1) |
| Q562R1 | Beta-actin-like protein 2 (Kappa-actin) |
| A5A3E0 | POTE ankyrin domain family member F (ANKRD26-like family C member 1B) (Chimeric POTE-actin protein) |
| P60174 | Triosephosphate isomerase (TIM) (EC 5.3.1.1) (Methylglyoxal synthase) (EC 4.2.3.3) (Triose-phosphate isomerase) |
| Q14019 | Coactosin-like protein |
| P23528 | Cofilin-1 (18 kDa phosphoprotein) (p18) (Cofilin, non-muscle isoform) |
| Q9H299 | SH3 domain-binding glutamic acid-rich-like protein 3 (SH3 domain-binding protein 1) (SH3BP-1) (TNF inhibitory protein B1) (TIP-B1) |
| P26038 | Moesin (Membrane-organizing extension spike protein) |
| P07737 | Profilin-1 (Epididymis tissue protein Li 184a) (Profilin I) |
| P62328 | Thymosin beta-4 (T beta-4) (Fx) [Cleaved into: Hemoregulatory peptide AcSDKP (Ac-Ser-Asp-Lys-Pro) (N-acetyl-SDKP) (AcSDKP) (Seraspenide)] |
| P12814 | Alpha-actinin-1 (Alpha-actinin cytoskeletal isoform) (F-actin cross-linking protein) (Non-muscle alpha-actinin-1) |
| Q15404 | Ras suppressor protein 1 (RSP-1) (Rsu-1) |
| P67936 | Tropomyosin alpha-4 chain (TM30p1) (Tropomyosin-4) |
| Q01518 | Adenylyl cyclase-associated protein 1 (CAP 1) |
| P27797 | Calreticulin (CRP55) (Calregulin) (Endoplasmic reticulum resident protein 60) (ERp60) (HACBP) (grp60) |
| P23284 | Peptidyl-prolyl cis-trans isomerase B (PPIase B) (EC 5.2.1.8) (CYP-S1) (Cyclophilin B) (Rotamase B) (S-cyclophilin) (SCYLP) |
| Q9ULV4 | Coronin-1C (Coronin-3) (hCRNN4) |
| O15144 | Actin-related protein 2/3 complex subunit 2 (Arp2/3 complex 34 kDa subunit) (p34-ARC) |
| O15511 | Actin-related protein 2/3 complex subunit 5 (Arp2/3 complex 16 kDa subunit) (p16-ARC) |
| P02788 | Lactotransferrin (Lactoferrin) (EC 3.4.21.-) (Growth-inhibiting protein 12) (Talalactoferrin) [Cleaved into: Lactoferricin-H (Lfcin-H); Kaliocin-1; Lactoferroxin-A; Lactoferroxin-B; Lactoferroxin-C] |
| P30086 | Phosphatidylethanolamine-binding protein 1 (PEBP-1) (HCNPpp) (Neuropolypeptide h3) (Prostatic-binding protein) (Raf kinase inhibitor protein) (RKIP) [Cleaved into: Hippocampal cholinergic neurostimulating peptide (HCNP)] |
| P31146 | Coronin-1A (Coronin-like protein A) (Clipin-A) (Coronin-like protein p57) (Tryptophan aspartate-containing coat protein) (TACO) |
| P05109 | Protein S100-A8 (Calgranulin-A) (Calprotectin L1L subunit) (Cystic fibrosis antigen) (CFAG) (Leukocyte L1 complex light chain) (Migration inhibitory factor-related protein 8) (MRP-8) (p8) (S100 calcium-binding protein A8) (Urinary stone protein band A) |
| P12429 | Annexin A3 (35-alpha calcimedin) (Annexin III) (Annexin-3) (Inositol 1,2-cyclic phosphate 2-phosphohydrolase) (Lipocortin III) (Placental anticoagulant protein III) (PAP-III) |
| P09211 | Glutathione S-transferase P (EC 2.5.1.18) (GST class-pi) (GSTP1-1) |
| P30740 | Leukocyte elastase inhibitor (LEI) (Monocyte/neutrophil elastase inhibitor) (EI) (M/NEI) (Peptidase inhibitor 2) (PI-2) (Serpin B1) |
| P25815 | Protein S100-P (Migration-inducing gene 9 protein) (MIG9) (Protein S100-E) (S100 calcium-binding protein P) |
| P04083 | Annexin A1 (Annexin I) (Annexin-1) (Calpactin II) (Calpactin-2) (Chromobindin-9) (Lipocortin I) (Phospholipase A2 inhibitory protein) (p35) [Cleaved into: Annexin Ac2-26] |
| P31949 | Protein S100-A11 (Calgizzarin) (Metastatic lymph node gene 70 protein) (MLN 70) (Protein S100-C) (S100 calcium-binding protein A11) [Cleaved into: Protein S100-A11, N-terminally processed] |
| P80511 | Protein S100-A12 (CGRP) (Calcium-binding protein in amniotic fluid 1) (CAAF1) (Calgranulin-C) (CAGC) (Extracellular newly identified RAGE-binding protein) (EN-RAGE) (Migration inhibitory factor-related protein 6) (MRP-6) (p6) (Neutrophil S100 protein) (S100 calcium-binding protein A12) [Cleaved into: Calcitermin] |
| P06744 | Glucose-6-phosphate isomerase (GPI) (EC 5.3.1.9) (Autocrine motility factor) (AMF) (Neuroleukin) (NLK) (Phosphoglucose isomerase) (PGI) (Phosphohexose isomerase) (PHI) (Sperm antigen 36) (SA-36) |
| P10599 | Thioredoxin (Trx) (ATL-derived factor) (ADF) (Surface-associated sulphydryl protein) (SASP) (allergen Hom s Trx) |
| P06702 | Protein S100-A9 (Calgranulin-B) (Calprotectin L1H subunit) (Leukocyte L1 complex heavy chain) (Migration inhibitory factor-related protein 14) (MRP-14) (p14) (S100 calcium-binding protein A9) |
| P06703 | Protein S100-A6 (Calcyclin) (Growth factor-inducible protein 2A9) (MLN 4) (Prolactin receptor-associated protein) (PRA) (S100 calcium-binding protein A6) |
| O00299 | Chloride intracellular channel protein 1 (Chloride channel ABP) (Nuclear chloride ion channel 27) (NCC27) (Regulatory nuclear chloride ion channel protein) (hRNCC) |
| P13796 | Plastin-2 (L-plastin) (LC64P) (Lymphocyte cytosolic protein 1) (LCP-1) |
| P14174 | Macrophage migration inhibitory factor (MIF) (EC 5.3.2.1) (Glycosylation-inhibiting factor) (GIF) (L-dopachrome isomerase) (L-dopachrome tautomerase) (EC 5.3.3.12) (Phenylpyruvate tautomerase) |
| P26447 | Protein S100-A4 (Calvasculin) (Metastasin) (Placental calcium-binding protein) (Protein Mts1) (S100 calcium-binding protein A4) |
| Q99497 | Parkinson disease protein 7 (Maillard deglycase) (Oncogene DJ1) (Parkinsonism-associated deglycase) (Protein DJ-1) (DJ-1) (Protein/nucleic acid deglycase DJ-1) (EC 3.1.2.-, EC 3.5.1.-, EC 3.5.1.124) |
| P37837 | Transaldolase (EC 2.2.1.2) |
| P09960 | Leukotriene A-4 hydrolase (LTA-4 hydrolase) (EC 3.3.2.6) (Leukotriene A(4) hydrolase) (Tripeptide aminopeptidase LTA4H) (EC 3.4.11.4) |
| P52209 | 6-phosphogluconate dehydrogenase, decarboxylating (EC 1.1.1.44) |
| P40121 | Macrophage-capping protein (Actin regulatory protein CAP-G) |
| P29401 | Transketolase (TK) (EC 2.2.1.1) |
| P00748 | Coagulation factor XII (EC 3.4.21.38) (Hageman factor) (HAF) [Cleaved into: Coagulation factor XIIa heavy chain; Beta-factor XIIa part 1; Coagulation factor XIIa light chain (Beta-factor XIIa part 2)] |

Note: Protein names derived from Uniprot.org

**Figure S2: Dissimilarity matrices for each of the 6 clusters**

*
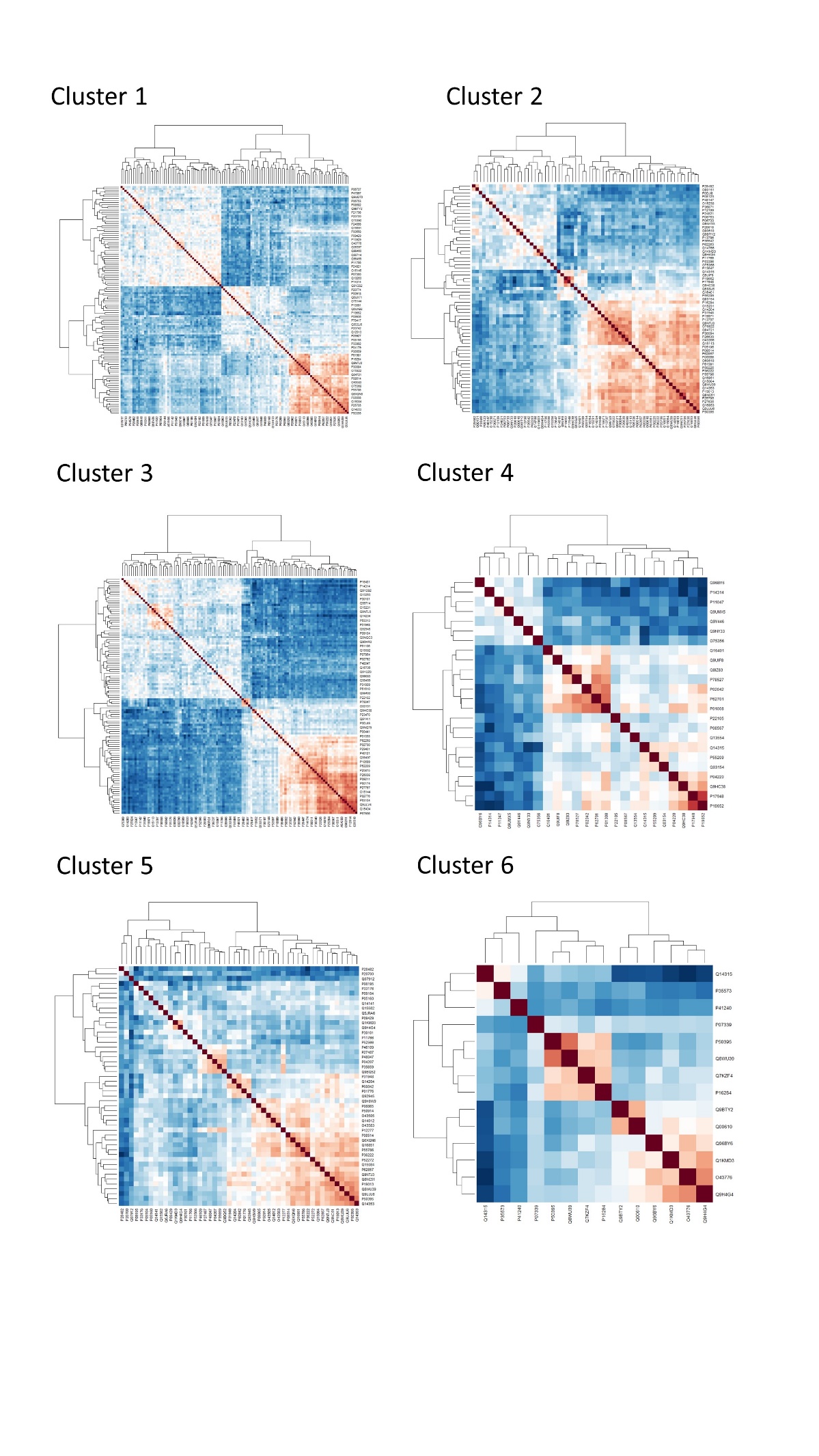
*

Table S5: Proteins and canonical pathways relating to the clusters 1A and 1B

|  | **Actin Binding** | **Cytoskeletal Protein Binding** | **Calcium Ion Binding** | **RAGE Receptor Binding** | **Protein Binding** |
| --- | --- | --- | --- | --- | --- |
| ACTN1 |  |  |  |  |  |
| ANXA1 |  |  |  |  |  |
| ANXA3 |  |  |  |  |  |
| ARPC2 |  |  |  |  |  |
| ARPC5 |  |  |  |  |  |
| CALR |  |  |  |  |  |
| CAP1 |  |  |  |  |  |
| CAPG |  |  |  |  |  |
| CFL1 |  |  |  |  |  |
| CORO1A |  |  |  |  |  |
| CORO1C |  |  |  |  |  |
| COTL1 |  |  |  |  |  |
| F12 |  |  |  |  |  |
| GAPDH |  |  |  |  |  |
| GPI |  |  |  |  |  |
| GSTP1 |  |  |  |  |  |
| LCP1 |  |  |  |  |  |
| MIF |  |  |  |  |  |
| MSN |  |  |  |  |  |
| PARK7 |  |  |  |  |  |
| PEBP1 |  |  |  |  |  |
| PF4 |  |  |  |  |  |
| PFN1 |  |  |  |  |  |
| PPIB |  |  |  |  |  |
| S100A11 |  |  |  |  |  |
| S100A12 |  |  |  |  |  |
| S100A4 |  |  |  |  |  |
| S100A6 |  |  |  |  |  |
| S100A8 |  |  |  |  |  |
| S100A9 |  |  |  |  |  |
| S100P |  |  |  |  |  |
| THBS1 |  |  |  |  |  |
| TKT |  |  |  |  |  |
| TMSB4X |  |  |  |  |  |
| TPI1 |  |  |  |  |  |
| TPM4 |  |  |  |  |  |
| YWHAZ |  |  |  |  |  |


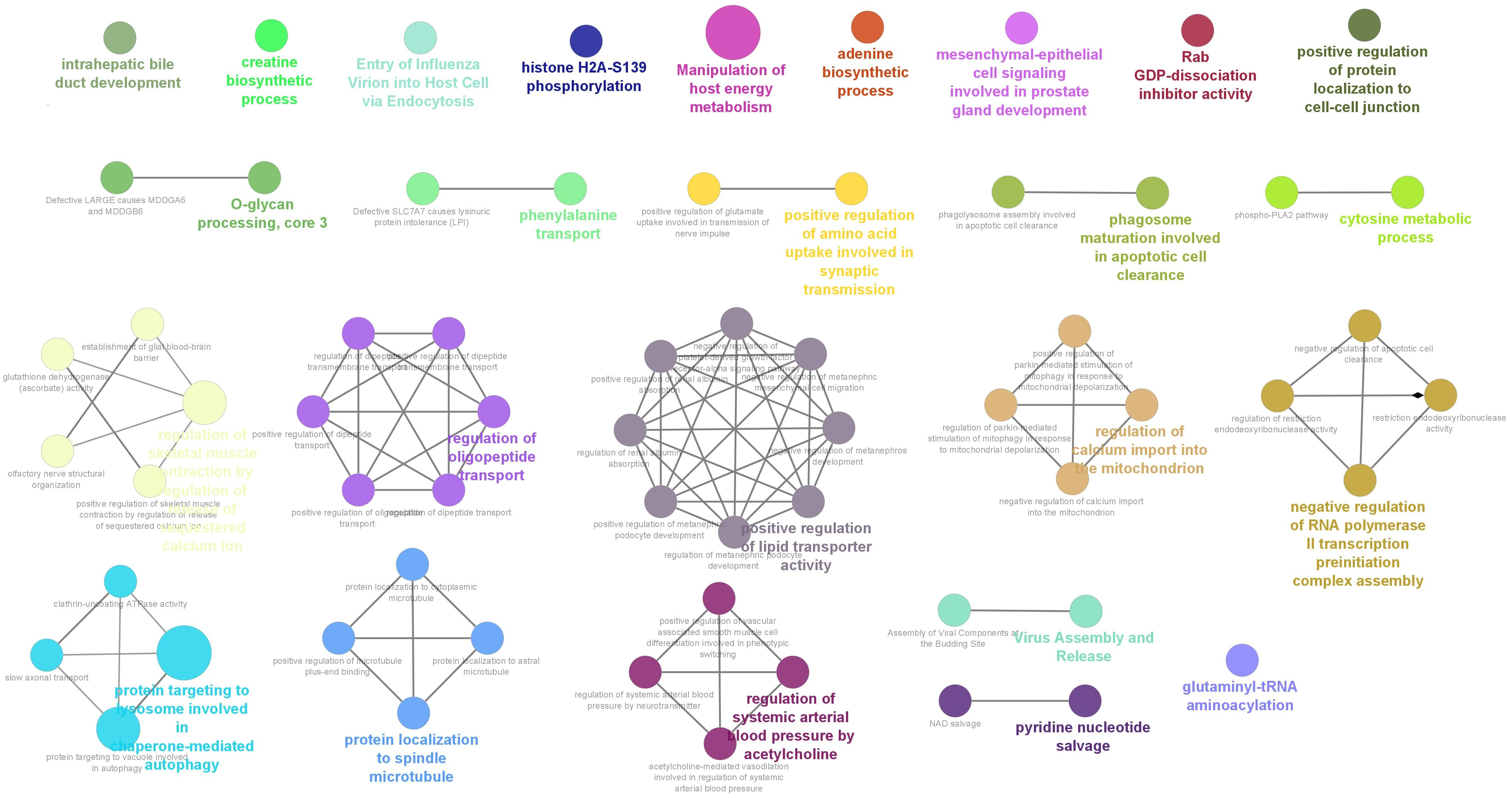


Fig S3. Gene Ontology of all proteins in cluster 1 utilising CLUEgo, a Cytoscape plugin for biological interpretation (4).

Table S6: Pathway analysis of the proteins which are increased or decreased in C6 compared to the rest of the cohort

| Ingenuity Canonical Pathways | -log(p-value) | Ratio | z-score | Molecules |
| --- | --- | --- | --- | --- |
| Hepatic Fibrosis Signaling Pathway | 2.75E+00 | 2.13E-02 | -2.121 | ICAM1, ITGB1, ITGB3, MAPK1, MYL6, PTK2, SOD2, TIMP1 |
| Tumor Microenvironment Pathway | 1.69E+00 | 2.27E-02 | -2 | ICAM1, ITGB3, MAPK1, TNC |
| Phospholipase C Signaling | 1.68E+00 | 1.88E-02 | -2 | AHNAK, ITGB1, ITGB3, MAPK1, MYL6 |
| Signaling by Rho Family GTPases | 3.11E+00 | 2.77E-02 | -1.633 | ARPC3, EZR, ITGB1, ITGB3, MAPK1, MYL6, PTK2 |
| Integrin Signaling | 2.77E+00 | 2.82E-02 | -1.633 | ARPC3, ITGA2B, ITGB1, ITGB3, MAPK1, PTK2 |
| Cdc42 Signaling | 3.20E+00 | 3.41E-02 | -1.342 | ARPC3, DIAPH1, ITGB1, ITGB3, MAPK1, MYL6 |
| PAK Signaling | 3.38E+00 | 4.72E-02 | -1 | ITGB1, ITGB3, MAPK1, MYL6, PTK2 |
| Rac Signaling | 3.12E+00 | 4.13E-02 | -1 | ARPC3, ITGB1, ITGB3, MAPK1, PTK2 |
| RhoA Signaling | 2.21E+00 | 3.25E-02 | -1 | ARPC3, EZR, MYL6, PTK2 |
| IL-8 Signaling | 1.51E+00 | 2.00E-02 | -1 | ICAM1, ITGB3, MAPK1, PTK2 |
| Neuroinflammation Signaling Pathway | 9.91E-01 | 1.33E-02 | -1 | HMGB1, ICAM1, MAPK1, SOD2 |
| Estrogen Receptor Signaling | 8.89E-01 | 1.22E-02 | -1 | ATP5F1A, MAPK1, MYL6, SOD2 |
| ILK Signaling | 3.03E+00 | 3.16E-02 | -0.816 | ITGB1, ITGB3, MAPK1, MYH9, MYL6, PTK2 |
| Actin Cytoskeleton Signaling | 5.08E+00 | 3.96E-02 | -0.707 | ARPC3, DIAPH1, EZR, ITGB1, ITGB3, MAPK1, MYH9, MYL6, PTK2 |
| Leukocyte Extravasation Signaling | 4.71E+00 | 4.15E-02 | -0.707 | EZR, ICAM1, ITGB1, MAPK1, MYL6, PECAM1, PTK2, TIMP1 |
| Ephrin Receptor Signaling | 3.04E+00 | 3.17E-02 | -0.447 | ACP1, ARPC3 ,ITGB1, ITGB3, MAPK1, PTK2 |
| MSP-RON Signaling In Cancer Cells Pathway | 2.92E+00 | 3.73E-02 | -0.447 | ITGB1, MAPK1, MST1, PTK2, YWHAG |
| Glioma Invasiveness Signaling | 3.04E+00 | 5.48E-02 | 0 | ITGB3, MAPK1, PTK2, TIMP1 |
| Cardiac Hypertrophy Signaling (Enhanced) | 7.70E-01 | 1.01E-02 | 0 | DIAPH1, ITGB1, ITGB3, MAPK1, PTK2 |
| Protein Kinase A Signaling | 2.59E+00 | 2.01E-02 | 0.378 | ACP1, MAPK1, MYL6, PTK2, PTPRG, PTPRJ, PYGL, YWHAG |
| Systemic Lupus Erythematosus In T Cell Signaling Pathway | 8.70E-01 | 1.20E-02 | 1 | EZR, ICOSLG/LOC102723996, MAPK1, PTK2 |

Supplementary methods references:

1. Liu Y, Buil A, Collins BC, et al. Quantitative variability of 342 plasma proteins in a human twin population. *Mol Syst Biol*. 2015;11(1):786. Published 2015 Feb 4. doi:10.15252/msb.20145728
2. Röst HL, Liu Y, D'Agostino G, Zanella M, Navarro P, Rosenberger G, et al. TRIC: an automated alignment strategy for reproducible protein quantification in targeted proteomics. Nat Methods. 2016;13(9):777-83.
3. Charrad M, Ghazzali N, Boiteau V, Niknafs A (2014). NbClust Package for Determining the Best Number of Clusters. R package version 2.0.3,

<http://CRAN.R-project.org/package=NbClust>

1. Bindea G, Mlecnik B, Hackl H, Charoentong P, Tosolini M, Kirilovsky A, Fridman WH, Pagès F, Trajanoski Z, Galon J. ClueGO: a Cytoscape plug-in to decipher functionally grouped gene ontology and pathway annotation networks. Bioinformatics. 2009 Apr 15;25(8):1091-3. doi: 10.1093/bioinformatics/btp101. Epub 2009 Feb 23. PMID: 19237447; PMCID: PMC2666812.
